# Molecular and phenotypic blueprint of human hematopoiesis links proliferation stress to stem cell aging

**DOI:** 10.1084/jem.20251805

**Published:** 2025-12-30

**Authors:** Emanuele Lettera, Luca Basso-Ricci, Edoardo Carsana, Kety Giannetti, Teresa Tavella, Luca Seffin, Giacomo Farina, Nicolò Gualandi, Pamela Quaranta, Elena Lo Furno, Guido Pacini, Lucrezia della Volpe, Kerstin B. Kaufmann, Laura Garcia-Prat, Raisa Jofra Hernandez, Alex Murison, Alicia G. Aguilar-Navarro, Stefano Beretta, Anastasia Conti, Eugenia Flores-Figueroa, Marco Ometti, Ivan Merelli, Alessandro Aiuti, Stephanie Z. Xie, Serena Scala, Raffaella Di Micco

**Affiliations:** 1 San Raffaele Telethon Institute for Gene Therapy (SR-TIGET), IRCCS Ospedale San Raffaele, Milan, Italy; 2 Università Vita-Salute San Raffaele, Milan, Italy; 3 University Health Network, Princess Margaret Cancer Centre, Toronto, Canada; 4 Unidad de Investigación Médica en Enfermedades Oncológicas, Centro Médico Nacional Siglo XXI, Instituto Mexicano del Seguro Social, Mexico City, Mexico; 5 Department of Orthopedic Surgery and Traumatology, IRCCS Ospedale San Raffaele, Milan, Italy; 6 Pediatric Immunohematology and Bone Marrow Transplantation Unit, IRCCS San Raffaele Scientific Institute, Milan, Italy; 7 Department of Medical Biophysics, University of Toronto, Toronto, Canada; 8 University School of Advanced Studies IUSS, Pavia, Italy

## Abstract

Hematopoietic stem/progenitor cells (HSPC) aging has long been associated with myeloid skewing, reduced clonal output, and impaired regenerative capacity, but quantitative immunophenotypic and functional analysis across the human lifespan has been lacking. Here, we provide a comprehensive phenotypic, transcriptional, and functional dissection of human hematopoiesis from youth to advanced age. Although primitive hematopoietic stem cell (HSC) numbers were stable during aging, overall cellularity declined, especially for erythroid and lymphoid lineages. HSPCs from older individuals exhibited repopulating frequencies comparable with those from younger donors in both primary and secondary xenografts; however, aged HSCs displayed impaired differentiation, chromatin and cell cycle dysregulation, and poor tolerance to activation-induced proliferative stress, resulting in DNA damage and senescence-like features after xenotransplantation. Importantly, imposing proliferative stress on young human HSPCs *in vivo* recapitulated key aging-associated phenotypic and functional declines. Together, our findings identify dysregulated activation responses as a defining feature of HSPC aging and establish proliferative stress–based xenotransplantation models as powerful platforms for investigating age-related hematopoietic dysfunctions.

## Teaser

Lettera et al. show that human hematopoiesis across aging is characterized by stable stem cell numbers and reduced erythroid/lymphoid output. Aged HSPCs retain engraftment yet exhibit impaired differentiation, altered chromatin states, inflammatory signatures, DNA damage, and senescence under stress. Proliferative stress in cord blood HSPCs recapitulates these defects, establishing a model for age-associated hematopoietic decline.

## Graphical Abstract

**Figure:**
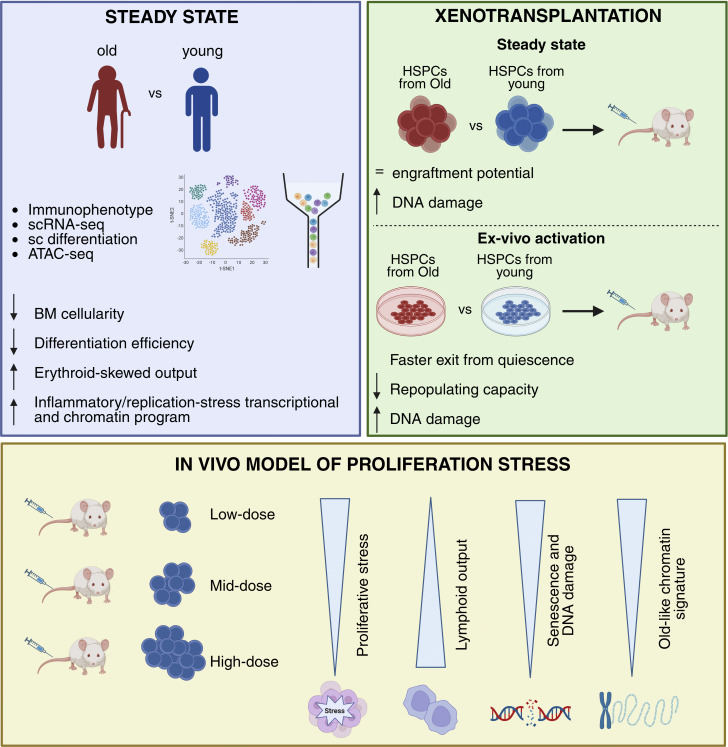

## Introduction

Age-associated changes in the hematopoietic stem and progenitor cells (HSPCs) compartment have been extensively unveiled in aged mice, where a marked myeloid skewing, impaired lymphoid and erythroid potential, and a decreased clonal output of hematopoietic stem cells (HSCs) have been reported (de Haan and Lazare, 2018; Morrison et al., 1996; Sudo et al., 2000). Mechanistically, changes in metabolic pathways, compromised maintenance of genome integrity, DNA replication stress, and epigenetic dysregulation have been linked to rapid exhaustion of the aging stem cell pool and impaired regeneration properties (Beerman et al., 2013; Beerman et al., 2014; Bernitz et al., 2016; Flach et al., 2014; Kirschner et al., 2017; Luchsinger et al., 2016; Morita et al., 2010; Oh et al., 2014; Rossi et al., 2005; Simsek et al., 2010; Sun et al., 2014; Walter et al., 2015; Wang et al., 2017; Wang et al., 2023; Young et al., 2021). Moreover, as transplantation itself imposes a strong proliferative stress on repopulating HSCs, aged murine HSCs have been shown to exhibit significantly diminished long-term repopulating potential relative to young HSCs, as well as reduced homing and engraftment capacity upon transplantation (Liang et al., 2005; Rossi et al., 2005). To date, it remains unclear to what degree these phenotypic observations on stem cell pool and lineage outputs in mouse settings as well as the underlying molecular principles are conserved during human hematopoietic aging. The functional decline of the lymphoid compartment, responsible for a defective adaptive immunity in old age, and the increased incidence of myeloproliferative disorders and hematopoietic myeloid malignancies in aged individuals (Henry et al., 2010; Jaiswal and Ebert, 2019; Signer et al., 2007; Vas et al., 2012; von Bonin et al., 2021) led to the common interpretation that, likewise in mice, human HSPC aging is associated with a prominent myeloid skewing (Chung and Park, 2017; Pang et al., 2017; van Galen et al., 2014). Moreover, there is evidence in humans that aging is associated with anemia (Stauder et al., 2018) and clonal hematopoiesis (CH) (Jaiswal and Ebert, 2019), which may act as additional cofactors in the development of myelodysplasia and hematopoietic malignancies (Avagyan and Zon, 2023; Shlush, 2018). Recent transcriptomic analyses of steady-state circulating HSPCs (cHSPCs) in the peripheral blood (PB) of healthy adults have revealed age-associated alterations in cHSPCs composition. Such age-related alterations are also associated with CH and a heightened risk of developing myelodysplastic syndromes (Furer et al., 2025). However, we and others have shown that bone marrow (BM) and PB HSPCs compartments differ in their phenotypic, transcriptomic, and functional properties under physiological conditions (Mende et al., 2022; Quaranta et al., 2024). Thus, it remains of paramount importance to investigate the impact of aging of HSPCs residing within the BM. A growing body of literature has uncovered the functional and molecular heterogeneity of human HSCs throughout life to maintain a constant blood supply under homeostatic steady-state conditions (Belluschi et al., 2018; García-Prat et al., 2021; Kaufmann et al., 2021; Laurenti and Göttgens, 2018; Milyavsky et al., 2010; Takayama et al., 2021; Zhang et al., 2022). Significant efforts led to the identification of surface markers and gene signatures to unambiguously define the subset of purified HSCs responsible for long-term outcomes upon transplantation (García-Prat et al., 2021; Laurenti et al., 2015; Notta et al., 2011; Notta et al., 2016; Takayama et al., 2021; Xie et al., 2021), and subsequent molecular studies performed in the context of aging had a clear focus on this primitive and rare cell subset. For instance, analysis of proliferation rate in highly purified HSCs has revealed altered responses of aged cells to mitogenic stimuli potentially contributing to hematopoietic dysfunctions upon transplant (Amoah et al., 2022; Hammond et al., 2023). However, it is worth noting that in several therapeutic applications for nonmalignant and malignant hematological diseases, a sizable fraction of a heterogenous population of progenitors and long-term HSCs (LT-HSCs) needs to be harvested, cultured *ex vivo* with early-acting cytokines, and reinfused in patients to maximize engraftment in the early phases after transplant. Furthermore, since transplantation outcomes are influenced by donor age (Artz, 2012; Duda et al., 2023; Jiang et al., 2022; Kollman et al., 2001; Mehta et al., 2023), characterizing the cellular and molecular landscape of both the mature and primitive HSPC compartments at steady state—as well as following pre-activation and transplantation—may provide valuable insights for improving HSPCs-based therapies in the elderly.

Here, we report a comprehensive quantitative characterization of blood cell subpopulations in BM and PB from a large healthy donor cohort across the human lifetime. We combined advanced genomics and *in vivo* primary and secondary transplantation assays to interrogate the functional contribution of proliferative stress to age-related changes in hematopoietic output and stem cell fitness. Overall, our work provides a blueprint of the HSPCs compartment upon aging and establishes a relevant xenotransplantation-based model for future mechanistic studies.

## Results

### A global reduction in lymphoid output and a steady HSCs content in BM during physiological aging

**Figure S1.**
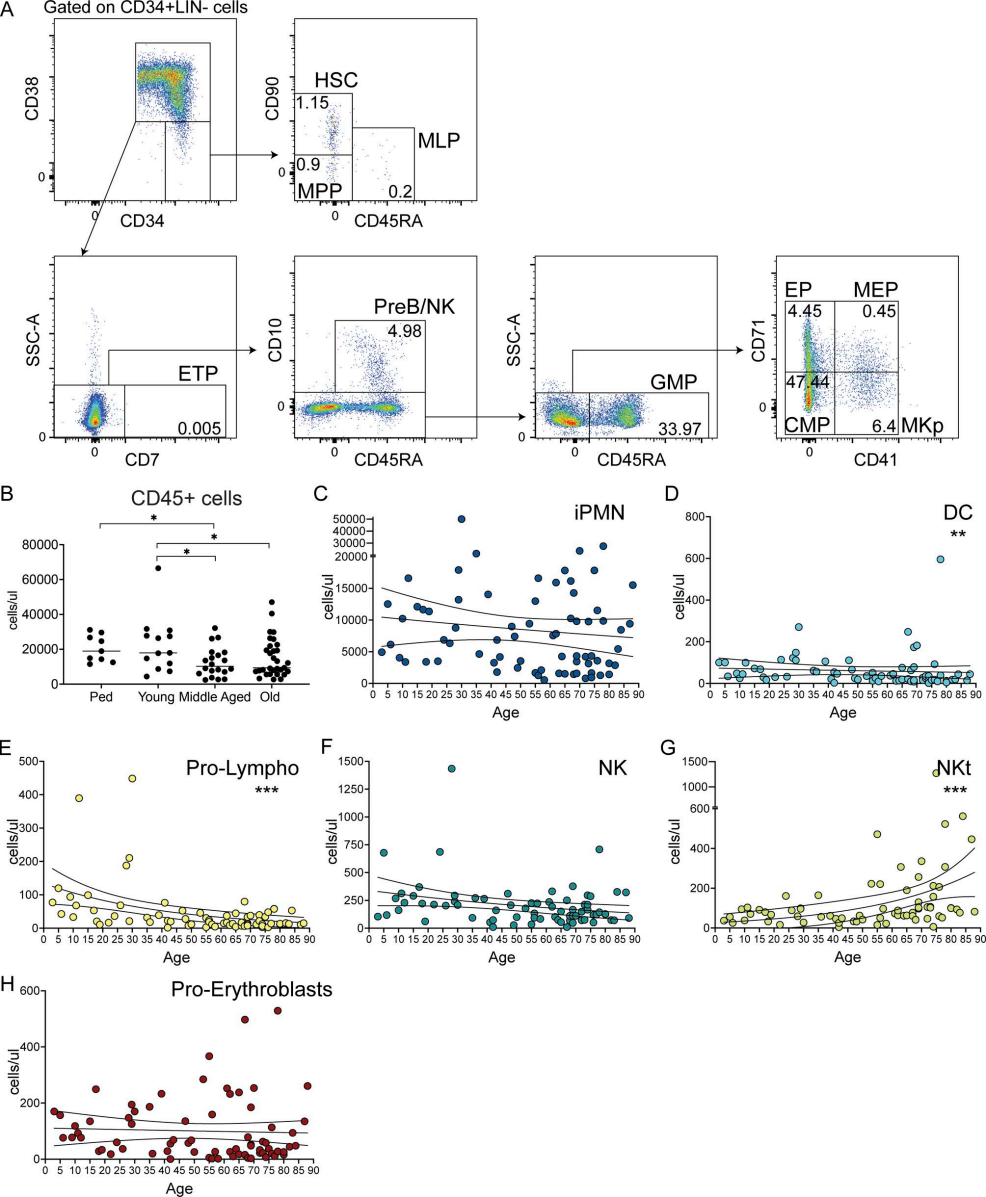
**Gating strategy for human HSPCs subsets and BM composition of healthy subjects across lifespan.** **(A)** Representative plots of CD34^+^LIN^−^ composition and gating strategy used for the identification of HSPCs subpopulations from a representative young donor. Numbers indicate the frequencies of each HSPCs subset on CD34^+^LIN^−^ cells. **(B)** Absolute quantification of CD45^+^ cells (expressed as cells/μl) from pediatric (<18 years, *n* = 9), young adult (18–40 years; *n* = 13), middle-aged (40–65 years; *n* = 20), and old (>65 years; *n* = 31) healthy subjects. Statistical test: Mann–Whitney; Ped vs. middle-aged, P = 0.021; Ped vs. old, P = 0.058; young vs. middle-aged, P = 0.0161; young vs. old, P = 0.026. **(C–H)** Correlation between the absolute count of each cell subtype (expressed as cells/μl) in the BM and the age of the 73 subjects analyzed. Statistical test: Spearman r; iPMN (immature polymorphonucleated cells), P = 0.176; DCs, P = 0.0015; Pro-lympho (lymphocytes progenitors), P = 0.0002; NK, P = 0.0743; NKt, P = 0.0002; proerythroblasts (erythroblast progenitors), P = 0.0861. ** < 0.01; *** < 0.001.

**Figure 1.**
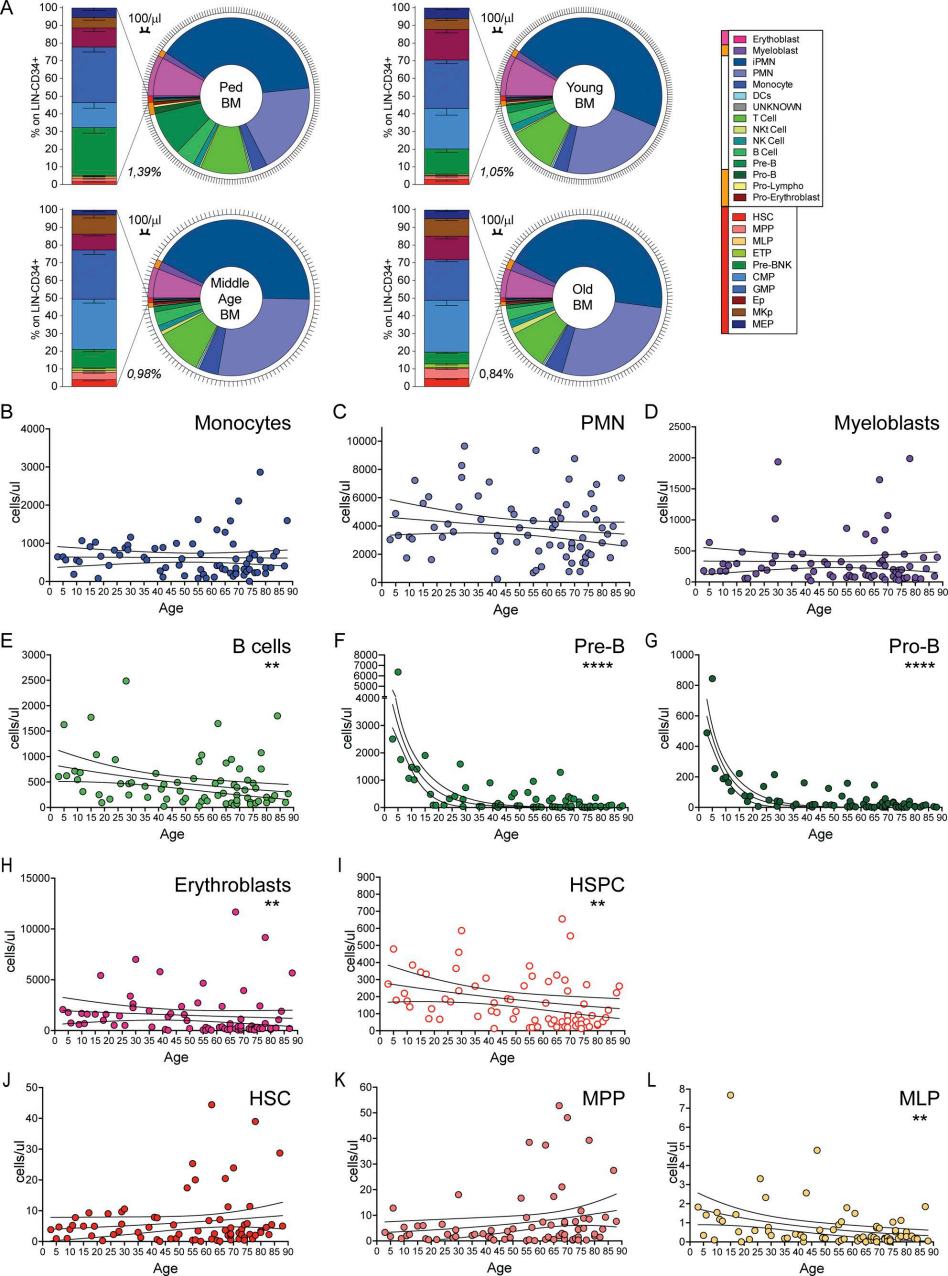
**BM composition of healthy subjects over aging. (A)** From top left to bottom right: Hematopoietic composition of BM samples from pediatric (<18 years, *n* = 9), young adult (18–40 years; *n* = 13), middle-aged (40–65 years; *n* = 20), and old (>65 years; *n* = 31) healthy subjects. Pie charts show the distribution of 25 hematopoietic subsets within the CD45^+^ gate. Black outer ticks indicate absolute count (100 cells/μl). Percentages indicate the frequency of the HSPCs population (CD34^+^ LIN^−^) on total CD45^+^ cells. The stacked bar graphs indicate the frequency of HSPCs subpopulations on LIN^−^CD34^+^ cells. **(B–L)** Correlation analysis of the absolute counts of each cell subtype (expressed as cells/μl) in the BM with the age of 73 subjects analyzed. Statistical test: Spearman r; monocytes, P = 0.2010; PMN (polymorphonucleated cells), P = 0.1799; myeloblasts, P = 0.1935; B cells, P = 0.0077; Pre-B, P < 0.0001; Pro-B, P < 0.0001; erythroblasts, P = 0.0067; HSPCs, P = 0.0017; HSCs, P = 0.9386; MPP (multipotent progenitors), P = 0.1432; MLP (multi-lymphoid progenitors), P = 0.0082. ** < 0.01; **** < 0.0001.

**Figure S2.**
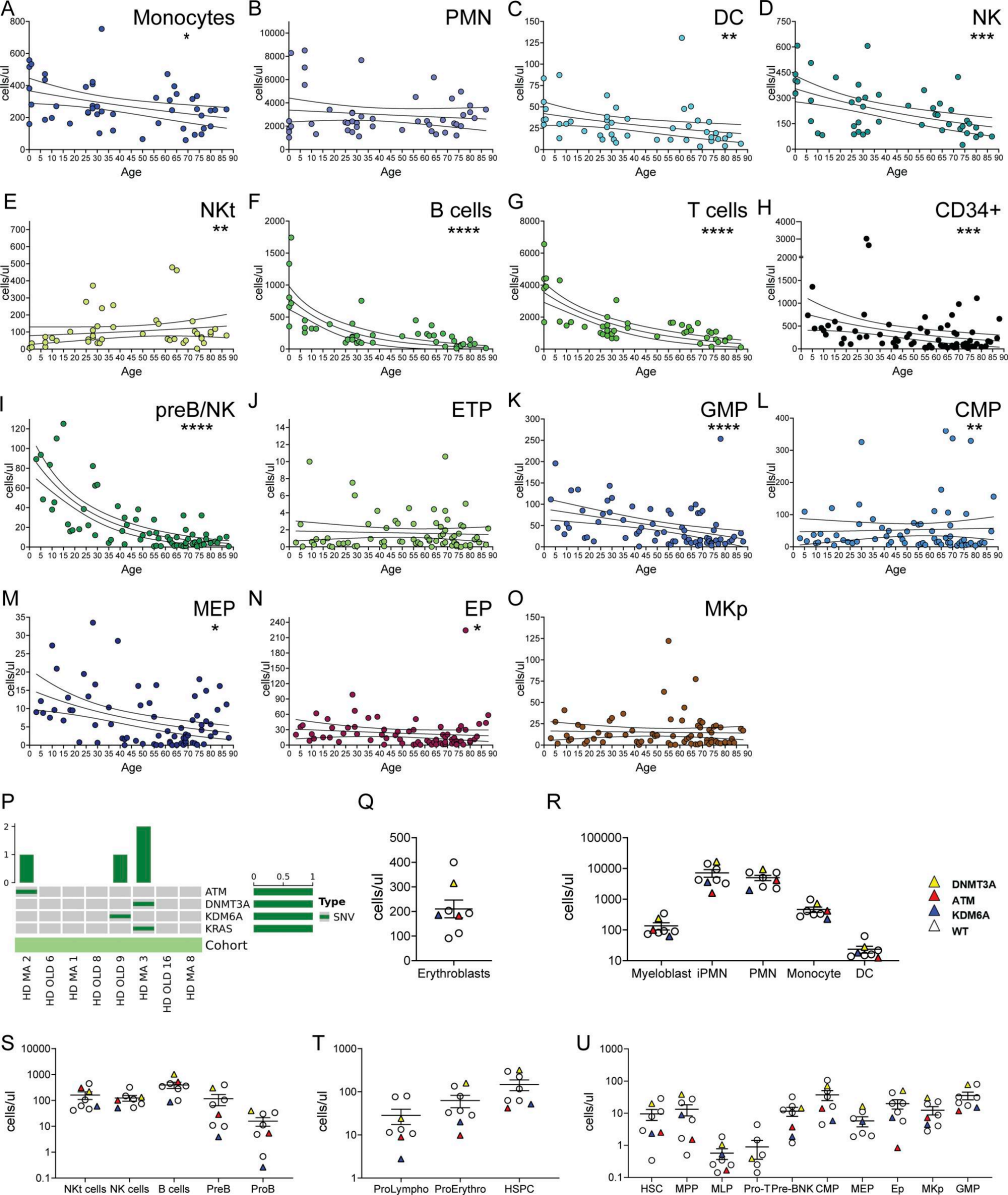
**Absolute counts of PB mature lineages and BM HSPCs subpopulations in healthy subjects across aging and their association with CH-related mutations.** **(A–G)** Correlation between the absolute count of each cell subtype (expressed as cells/μl) in the PB and the age of the 56 subjects analyzed. Statistical test: Spearman r; monocytes, P = 0.011; PMN (polymorphonucleated cells), P = 0.491; DCs, P = 0.0011; NK, P = 0.0002; NKt P = 0.0086; B cells, P < 0.0001; T cells, P < 0.0001. **(H–O)** Correlation between the absolute count of each cell subtype (expressed as cells/μl) in the BM and the age of the 73 subjects analyzed. Statistical test: Spearman r; CD34^+^ cells, P = 0.0002; PreB/NK, P < 0.0001; ETP (early T progenitors), P = 0.806; GMP (granulocyte-monocyte progenitors), P < 0.0001; CMP (common myeloid progenitors), P = 0.005; MEP (megakaryocyte-erythrocyte progenitors), P = 0.014; EP (erythrocyte progenitors), P = 0.014; MKp, (megakaryocyte progenitors), P = 0.088. **(P)** CH-associated mutations analysis in aged BM samples (*n* = 8) via whole-exome sequencing. (**Q–U)** Absolute quantification of each cell subtype (expressed as cells/μl) in the BM of WT (*n* = 5) and CH-associated mutations (*n* = 3) donors. * < 0.05; ** < 0.01; *** < 0.001; **** < 0.0001.

To dissect the changes in hematopoietic cell composition during aging, we analyzed 73 BM samples from pediatric (0–18 years, *n* = 9), young adult (18–30 years, *n* = 13), middle-aged (40–65 years, *n* = 20), and old (>65 years, *n* = 31) healthy individuals. As an additional and complementary dataset, we collected 56 PB samples from 12 pediatric, 17 young, 8 middle-aged, and 19 old subjects (Table S1). By means of multiparametric flow cytometry analysis (Basso-Ricci et al., 2017), we found a significant decrease in BM cellularity and in the frequency of the HSPCs compartment during aging (Fig. S1, A and B; and Fig. 1 A). Myeloid compartment remained stable over aging, since we did not observe any correlations between donors’ age and monocytes, immature granulocytes (iPMN), mature granulocytes (PMN), and myeloblasts cell counts, with the only exception of dendritic cells (DC), whose number decreased over time (Fig. 1, B–D; and Fig. S1, C and D). We also found a gradual decrease of lymphocyte progenitor (Fig. S1 E) and B cell count during aging, while a dramatic drop of B cell precursors (PreB) and B cell progenitors was observed as early as 30 years of age (Fig. 1, E–G). BM-resident natural killer (NK) cells showed a slight reduction, while NK T (NKt) lymphocytes increased during aging (Fig. S1, F and G). Our analyses also revealed a decline in the number of erythroblasts and proerythroblasts with age (Fig. 1 H and Fig. S1 H), in line with the severe anemia reported in elderly subjects (Stauder et al., 2018). Similar changes in mature hematopoietic cell composition were also detected in the PB of the analyzed subjects, with marked reduction mainly in the absolute count of B, NK, and T lymphocytes (Fig. S2, A–G).

We next evaluated the changes occurring within the most undifferentiated hematopoietic compartment and found reduced cell counts of both CD34^+^ cells as well as of CD34^+^LIN^−^ cells (defining the HSPCs population) (Fig. 1 I and Fig. S2 H). Of note, the number of primitive HSCs and MPP subsets was stable during aging (Fig. 1, J and K), while a prominent decline was observed in lymphoid (MLP and PreB/NK), myeloid (CMP and GMP), and erythroid (EP and MEP) committed progenitors, except for early T progenitors and megakaryocyte progenitors, which remained stable over aging (Fig. 1 L and Fig. S2, I–O). To evaluate the impact of CH on the observed immunophenotypic changes, we sequenced via whole-exome sequencing, 8/31 aged BM samples in the old cohort for whom material was available and detected CH-associated mutations in *DNMT3A/KRAS*, *KDM6A*, and *ATM* genes in 3/8 donors with a variant allele frequency (VAF) <0.2. Nevertheless, the hematopoietic cell counts of the subjects with CH mutations were consistent with the counts measured in individuals with no CH (Fig. S2, P–U).

**Figure S3.**
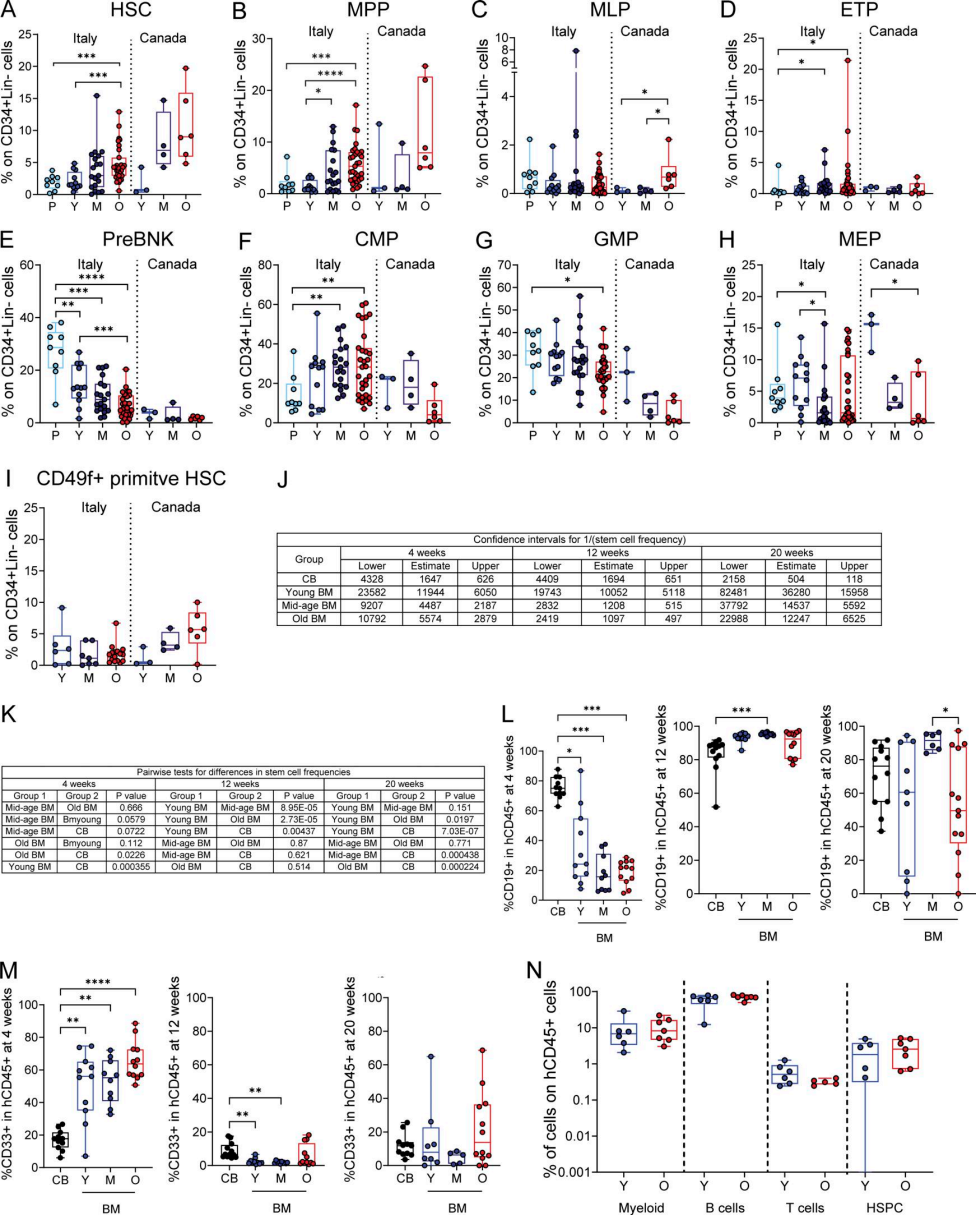
**Age-dependent remodeling of human HSPCs compartment, stem cell frequency and functional output across distinct age cohorts.** **(A–H)** Frequencies of distinct HSPCs subpopulations within CD34^+^LIN^−^ compartment in healthy donors from different ages in the Italian cohort (P = Pediatric <18 years, *n* = 9; Y = young 18–35 years, *n* = 12; M = middle-aged 40–60 years, *n* = 20; O = old >65 years, *n* = 31) and in the North American (Canada) cohort (Y: *n* = 3; M: *n* = 4; O: *n* = 6). Statistical test: Mann–Whitney; HSCs Italy: P vs. O, P = 0.0004; Y vs. O, P = 0.0007. MPP Italy: P vs. O, P = 0.0008; Y vs. M, P = 0.0392; Y vs. O, P < 0.0001. MLP Canada: Y vs. O, P = 0.0476; M vs. O, P = 0.019. ETP Italy: P vs. M, P = 0.0186; P vs. O, P = 0.0303. PreB/NK Italy: P vs. Y, P = 0.0073; P vs. M, P = 0.0002; P vs. O, P < 0.0001; Y vs. O, P = 0.0009. CMP Italy: P vs. M, P = 0.0016; P vs. O, P = 0.0027. GMP Italy: P vs. O, P = 0.0146. MEP Italy: P vs. M, P = 0.0211; Y vs. M, P = 0.0157; MEP Canada: Y vs. O, P = 0.024. **(I)** Frequencies of CD49f^+^-primitive HSCs (defined as CD34^+^CD19^−^CD38^−^CD45RA-CD90^+^CD49f^+^ cells) within CD34^+^LIN^−^ compartment in healthy donors from different ages in the Italian (Y: *n* = 6; M: *n* = 7; O: *n* = 16) and in the North American (Canada) cohort (Y: *n* = 3; M: *n* = 4; O: *n* = 6). **(J and K)** Tables showing the confidence intervals for 1/stem cell frequency and P values from pairwise test for differences in the stem cell frequencies for the indicated sample groups as calculated by ELDA. Referring to Fig. 2. (**L and M)** Box plots showing human CD19^+^ and CD33^+^ cell frequencies in the murine BM of mice transplanted with 60,000 cells for CB and BM samples at 4–12 and 20 wk. Statistical test: Mann–Whitney; B cells, 4 wk: CB vs. Y, P = 0.0287; CB vs. M, P = 0.0001; CB vs. O, P = 0.0001. 12 wk: CB vs. M, P = 0.0002. 20 wk: M vs. O, P = 0.0338. Myeloid cells, 4 wk: CB vs. Y, P = 0.007; CB vs. M, P = 0.0065; CB vs. O, P < 0.0001; 12 wk: CB vs. Y, P = 0.007; CB vs. M, P = 0.0065. **(N)** Box plot showing different human subpopulations frequencies in the murine BM in secondary recipients at 12 wk. * < 0.05; ** < 0.01; *** < 0.001; **** < 0.0001.

To exclude any potential geographic or collection bias that might account for the observed changes among donors of different age groups, we validated our findings in a North American cohort of 13 donors (age range, 21–83 years). We found consistent changes in HSPCs subsets between the two cohorts of healthy subjects (Fig. S3, A–I). Of note, we observed an increased frequency of primitive subpopulations within the HSPCs compartment during aging (Fig. S3, A and B), in line with previous reports (Pang et al., 2011), while the frequency of LT-HSCs (defined as CD34^+^CD19^−^CD38^−^CD45RA-CD90^+^CD49f^+^ cells) remained stable across ages (Fig. S3 I).

Altogether, our data indicate that, despite the stable HSCs and MPP cell counts, lineage-committed HSPCs progenitors and the overall BM cellularity are severely reduced over aging. This latter observation is the result of a sharp reduction of the lymphoid cell production (especially in the B cell compartment), while myeloid cell counts remained steady across aging.

### Human HSPCs repopulating potential in xenotransplantation assays is comparable across age

**Figure 2.**
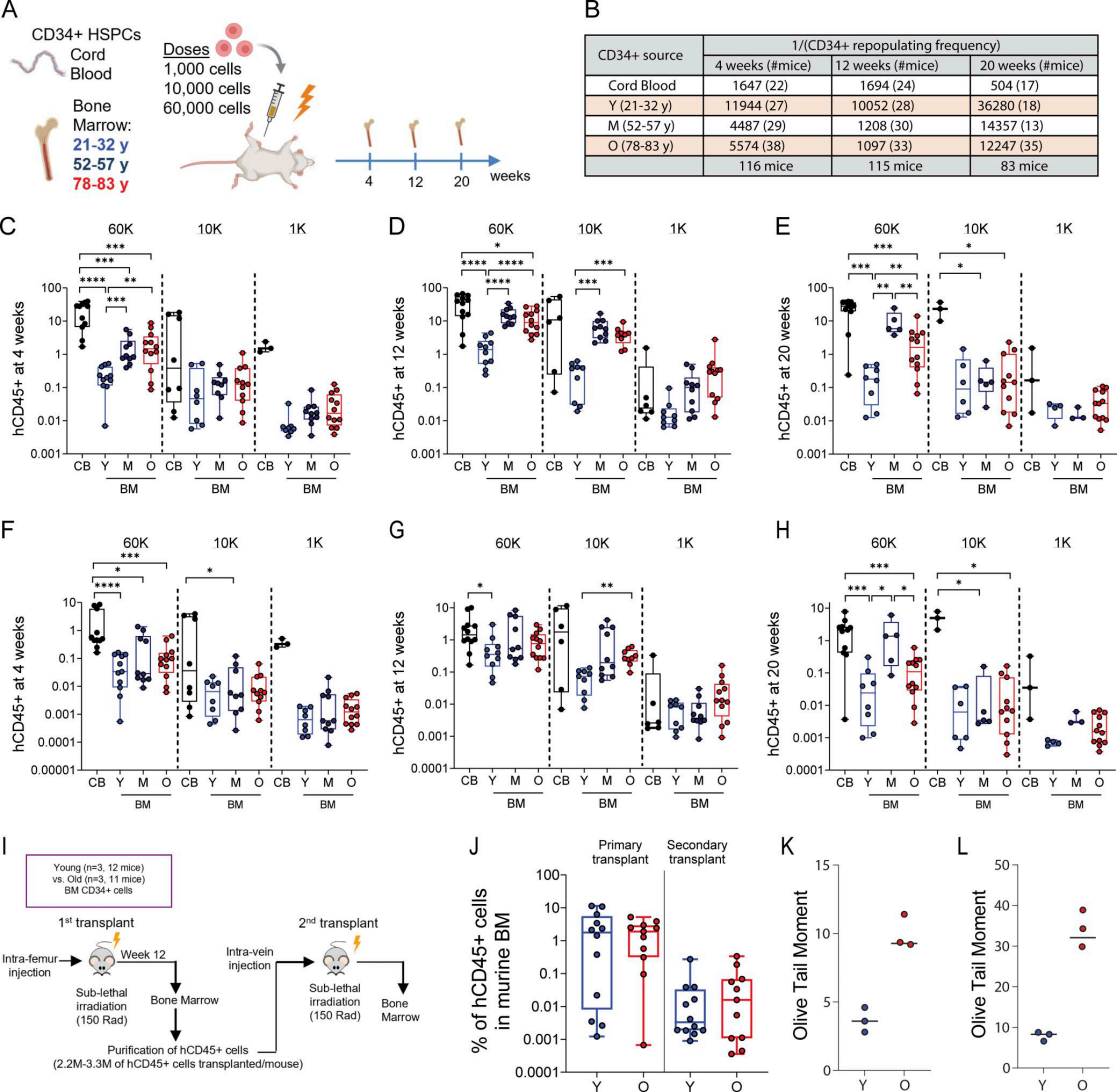
**Limiting dilution xenotransplantation assays of uncultured HSPCs show comparable frequency of repopulating cells in aged HSPCs compared with young HSPCs. (A)** Experimental scheme using three cell doses in xenotransplantation time course experiments (*n* = 4) where human CD45^+^ engraftment and lineage in the BM of NSG (NOD.Cg *Prkdc*scid*Il2rg*tm1Wjl/SzJ) mice were assessed at 4, 12, and 20 wk for the indicated human HSPCs cell sources (number of donors for each source: CB = 4; young BM = 5; middle-aged BM = 3; old BM = 5). **(B)** The CD34^+^ repopulating cell frequency for 4, 12, and 20 wk for CB and BM samples from indicated age groups. The number reported in brackets in the table display the total number of transplanted mice for each source or age group considering all cell doses at each time point. **(C–E)** Box plots showing human CD45^+^ cell frequencies in the murine BM in mice transplanted with 60,000 (60K), 10,000 (10K), and 1,000 (1K) HSPCs for CB and BM samples at 4 (C), 12 (D), and 20 (E) wk. Transplanted mice with engraftment below 0.05% in the murine BM were considered not engrafted. Statistical test: Mann–Whitney; 4 wk, 60K: CB vs. Y, P < 0.0001; CB vs. M, P = 0.0001; CB vs. O, P = 0.0002; Y vs. M, P = 0.0001; Y vs. O, P = 0.009. 12 wk, 60K: CB vs. Y, P < 0.0001; CB vs. O, P = 0.028; Y vs. M, P < 0.0001; Y vs. O, P < 0.0001. 10K: Y vs. M, P = 0.0002; Y vs. O, P = 0.0004; 20 wk, 60K: CB vs. Y, P = 0.0002; CB vs. O, P = 0.0005; Y vs. M, P = 0.0016; Y vs. O, P = 0.0055; M vs. O, P = 0.0094. 10K: CB vs. M, P = 0.0357; CB vs. O, P = 0.0167. **(F–H)** Box plots showing human CD45^+^ cell frequencies in the murine BM in mice transplanted with 60K, 10K, and 1K HSPCs for CB and BM samples at 4 (F), 12 (G), and 20 (H) wk normalized on the HSC+MPP content of the infused cells. Statistical test: Mann–Whitney; 4 wk, 60K: CB vs. Y, P < 0.0001; CB vs. M, P = 0.0295; CB vs. O, P = 0.0005. 10K: CB vs. M, P = 0.0426; 12 wk, 60K: CB vs. Y, P = 0.0206. 10K: Y vs. O, P = 0.0028. 20 wk, 60K: CB vs. Y, P = 0.0008; CB vs. O, P = 0.0007; Y vs. M, P = 0.0109; M vs. O, P = 0.0268. 10K: CB vs. M, P = 0.0357; CB vs. O, P = 0.0167. **(I)** Experimental scheme for secondary transplant. CD34^+^ cells isolated from three young and three old donors were intrafemorally transplanted in 23 primary NSG (NOD.Cg *Prkdc*scid*Il2rg*tm1Wjl/SzJ) recipients (each donor into three to four mice). At 12 wk after transplant, human CD45^+^ cells from the BM of primary mice were collected and transplanted into 23 secondary recipients. The murine BM of the secondary recipients was analyzed at 12 wk. Transplanted mice with engraftment below 0.05% (for primary transplants) and 0.005% (for secondary transplants) in the murine BM were considered not engrafted. The threshold for secondary mice was set on not transplanted control mice. Not engrafted mice were plotted in the graphs but excluded from the statistical analyses. **(J)** Box plots showing human CD45^+^ cell frequencies in the murine BM in primary and secondary recipients at 12 wk, normalized on the content of HSC+MPP infused. **(K and L)** Olive Tail Moment average values of neutral (K) or alkaline (L) comet experiments conducted in CD34^+^ isolated at 12 wk after secondary transplantation of CD34^+^ cells from young and old donors (CD34^+^ isolated from the mice transplanted with the same donor were pooled). * < 0.05; ** < 0.01; *** < 0.001; **** < 0.0001.

We next undertook a comprehensive limiting dilution xenotransplantation study to quantitatively measure engraftment of young (21–32 years, *n* = 5), middle-aged (52–57 years, *n* = 3), and old (78–83 years, *n* = 5) BM CD34^+^ cells relative to the neonatal Cord Blood-derived (CB-derived) CD34^+^ cells (*n* = 4) using 314 immunodeficient nonobese diabetic (NOD)-severe combined immunodeficiency-IL2R-γ^−/−^ (NSG) mice (Fig. 2 A). Three cell doses (60,000, 10,000, and 1,000 cells) were selected based on a prior study to measure CB functional HSC frequency (Laurenti et al., 2015). Human CD45 engraftment levels were measured at 4, 12, and 20 wk after transplantation and used to calculate the 1/CD34^+^ repopulating cell frequency in all four sources of human HSPCs (Fig. 2, B–E; Table S2; and Fig. S3, J and K). As expected, CB repopulating cell frequency was superior to BM sources at 4 and 20 wk after transplantation, while we observed a comparable repopulation frequency among CB, BM middle-aged, and BM old HSPCs at 12 wk (Wang et al., 1997) (Fig. 2 B; and Fig. S3, J and K). CD34^+^ repopulating cell frequency kinetics was found to undergo dynamic changes across time, with BM sources peaking at 12 wk and dropping to the lowest frequency at 20 wk. By contrast, the stem cell frequency for CB was highest at 20 wk (1/504 CD34^+^ cells, Fig. 2 B and Fig. S3 J). Surprisingly, middle-aged and old BM HSPCs had a significantly higher CD34^+^ repopulating cell frequency at 12 wk (Fig. 2 B) and showed superior engraftment relative to young BM HSPCs at the 60,000-cell dose for all time points and at the 10,000-cell dose for 12 wk (Fig. 2, C–E). Additionally, at 4 wk, human cell grafts contained lower B cells but a higher fraction of myeloid cells in all the mice transplanted with BM-CD34^+^ cells with respect to CB, independently from the age of the BM donors, likely reflecting a distinct proportion of repopulating hematopoietic progenitors in the two cell sources (Fig. S3, L and M). To account for the differences in HSC+MPP frequency in old vs. young HSPCs (Figs. 1, S1, S2, and S3), we normalized the human content in the BM of transplanted mice for the number of infused HSC+MPP in young, middle-aged, and old experimental groups (Fig. 2, F–H). This analysis showed that although CB engraftment remained overall higher than that of adult BM sources, young and old BM HSPCs exhibited comparable engraftment capacity at 4 and 20 wk, with old cells displaying a higher engraftment capacity at the 12-wk time point for the 10,000-cell dose (Fig. 2, F–H).

Additionally, to further assess the self-renewal capacity of LT-HSCs from old vs. young donors *in vivo*, we performed secondary transplants from young (*n* = 3) or old (*n* = 3) donors in 23 NSG mice (each donor into three to four mice) (Fig. 2 I). The BM of secondary recipients was analyzed after 12 wk, and data normalized on HSC+MPP content in the infused HSPCs were reported. Strikingly, we found no differences in the human cell engraftment and lineage output both in primary and secondary recipients when transplanting young and old HSPCs (Fig. 2 J and Fig. S3 N). However, we measured higher presence of both single- and double-stranded DNA breaks in old vs. young HSPCs isolated from secondary transplanted mice, implying that old LT-HSCs have reduced capability of counteracting DNA damage imposed by xenotransplantation (Fig. 2, K and L).

Overall, these data indicate that human-aged (HSCs) have a similar *in vivo* long-term repopulating capacity but increased sensitivity to transplantation stress than their young counterparts.

### Aged LT-HSCs have reduced differentiation potential associated with transcriptomic and chromatin accessibility alterations

**Figure 3.**
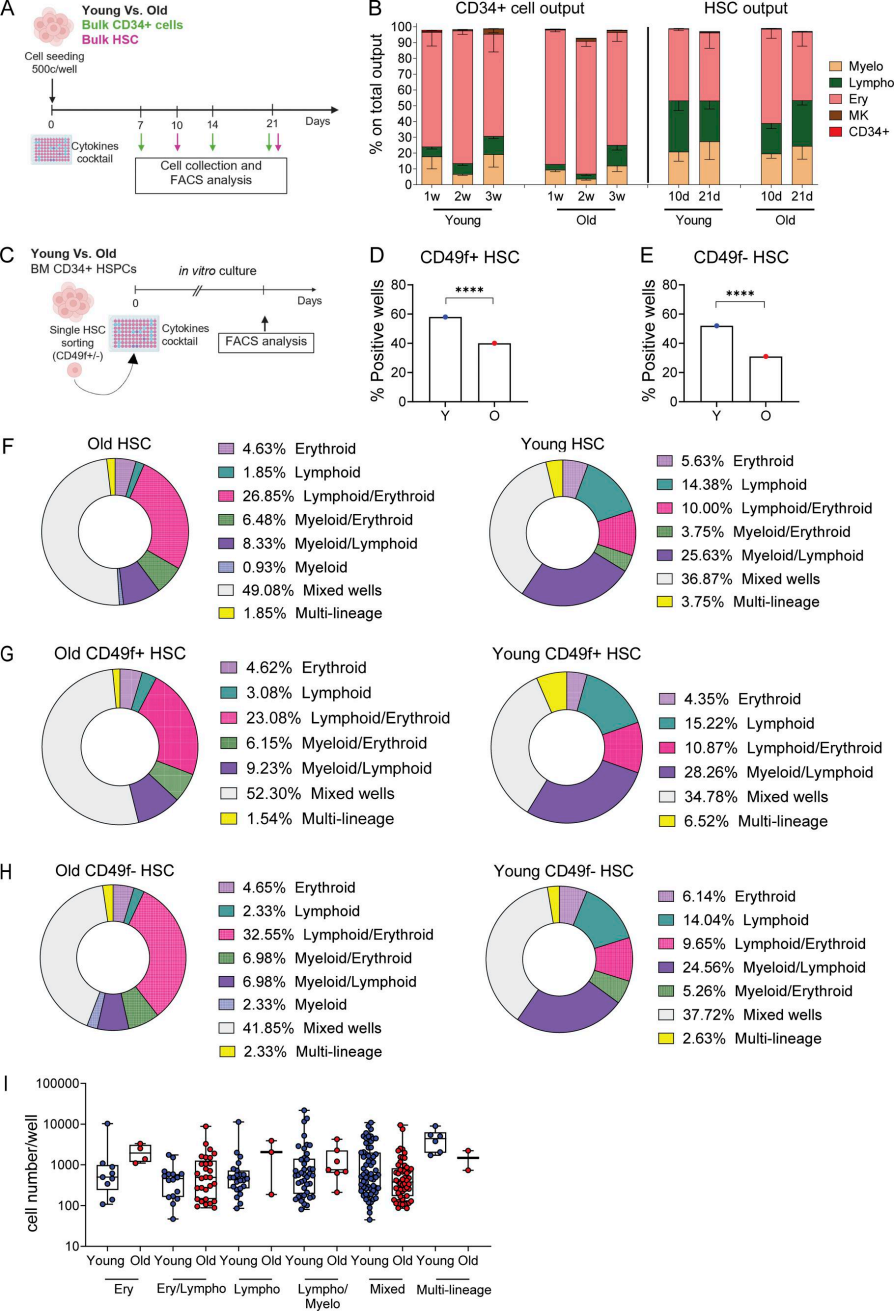
**Single-cell functional analyses of aged LT-HSCs. (A)** Experimental scheme for *in vitro* multi-lineage differentiation assay on bulk CD34^+^ and HSCs (500 cells/well) from young (*n* = 3) and old (*n* = 3) donors. **(B)** Differentiation output of bulk CD34^+^ and HSCs from young and old donors at different time points. **(C)** Experimental scheme for *in vitro* multi-lineage differentiation assay on single CD49f^+/−^ HSCs (100 cells/donor) from young (*n* = 3) and old (*n* = 3) donors. **(D and E)** Differentiation efficiency of single CD49f^+^ HSCs (D) and CD49f- HSCs (E) from young and old donors. Pearson’s Chi-squared test with Yates’ continuity correction was used to measure statistical significance of the observed differences. CD49f^+^: Y vs. O, P < 0.0001; CD49f^−^: Y vs. O, P < 0.0001. **(F–H)** Pie charts representing the lineage scores of young and old total HSCs (F), CD49f^+^ HSCs (G), and CD49f^−^ HSCs (H), based on the most abundant differentiation outputs toward lymphoid, myeloid, erythroid, and megakaryocyte lineages detected at the end of the single-cell *in vitro* differentiation assay. Cells showing differentiation toward all the four lineages were scored as “multi-lineage”. **(I)** Graph showing the absolute cell count of single HSCs clones output scored according to their lineage production. **** < 0.0001.

Given the reduced support to human myeloid and erythroid production of NSG mice and the restriction to the B cell lymphoid output in the human graft, we investigated the differentiation capacity of human CD34^+^ cells as well as purified HSCs from young and old donors in an optimized *in vitro* stroma-free multi-lineage assay, allowing the differentiation toward lymphoid, myeloid, erythroid, and megakaryocyte lineages (Fig. 3 A) (Quaranta et al., 2024; Scala et al., 2023). Our results showed that both bulk CD34^+^ cells and HSCs from young and old donors display similar differentiation output in culture (Fig. 3 B). By performing the same *in vitro* differentiation assay on single, individual HSCs, stained with CD49f marker for index sorting analyses (Fig. 3 C), we found that the differentiation efficiency of both CD49f^+^ and CD49f^−^ aged HSCs was lower with respect to their younger counterparts (Fig. 3, D and E). We also scored the differentiation output of the single colonies and observed an overall decrease of lymphoid-only or myeloid-lympho clonal outputs in aged HSCs, while the proportion of lymphoid-erythroid colonies was increased (Fig. 3 F). Additionally, we detected uni-myeloid colonies only in old HSCs, albeit at low frequency. The same trends were also observed when dissecting LT-HSC according to the expression of CD49f surface marker (Fig. 3, G and H). Of note, we measured an increased fraction of clones showing multi-lineage production in young vs*.* old LT-HSCs (6.52% vs. 1.54%), suggesting a reduced differentiation potential of individual aged LT-HSCs and an increased primitiveness of young LT-HSCs. Along this line, we did not detect any differences in the number of cells produced by old and young LT-HSCs clones, except for a trend of higher cellularity in young vs. old multi-lineage LT-HSCs clones (Fig. 3 I).

**Figure 4.**
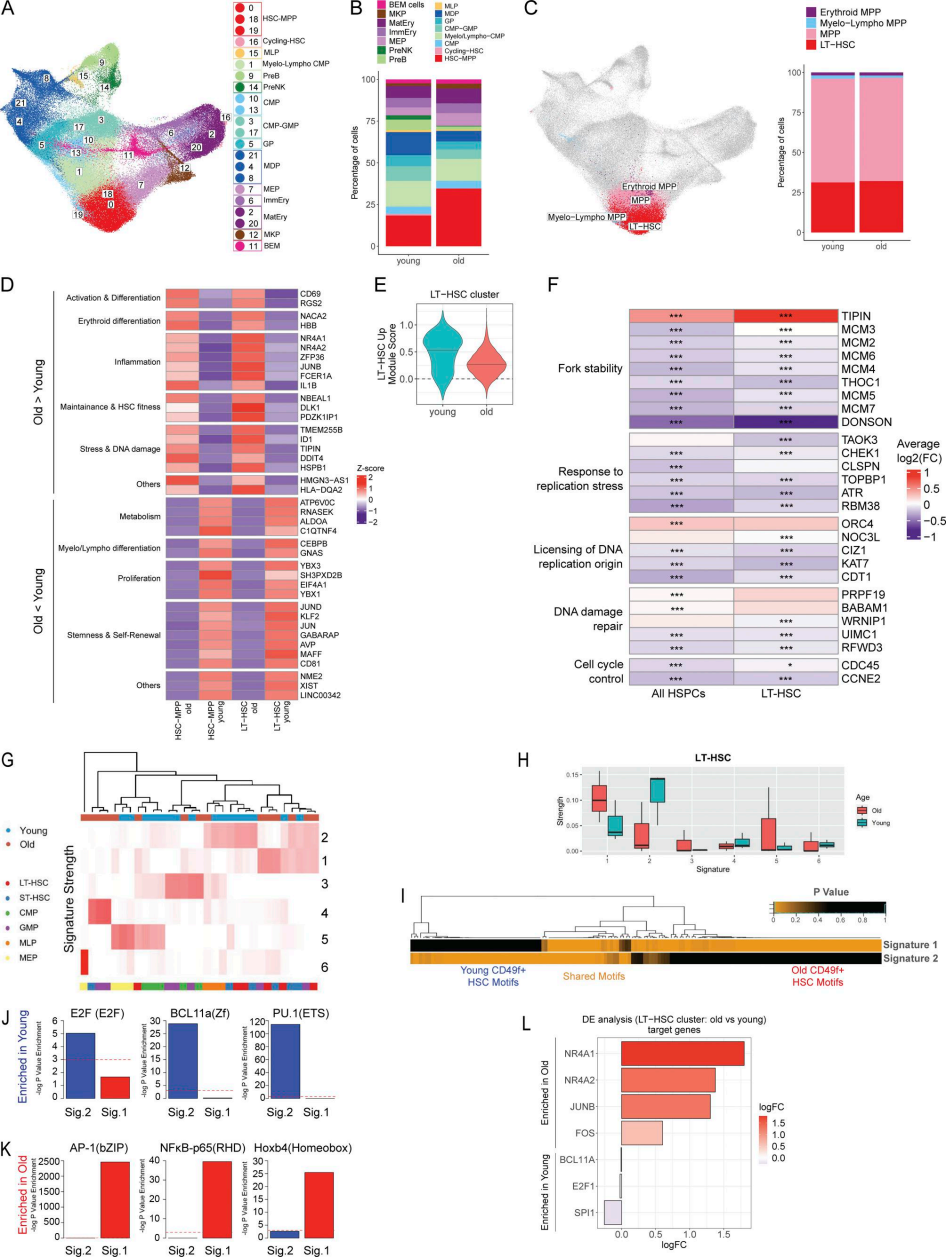
**Single-cell transcriptomic and epigenomic analyses of aged LT-HSCs. (A)** UMAP embedding showing 21 Seurat clusters identified after unsupervised clustering. The legend on the right shows the annotation of the single clusters. **(B)** Stacked bar graph showing the distribution of the transcriptional annotated clusters in young and old HSPCs. **(C)** On the left, UMAP embedding showing four annotated Seurat subclusters identified after unsupervised clustering of cluster 0. On the right, stacked bar graph showing the distribution of the four transcriptional subclusters in young and old HSCs. **(D)** Heatmap showing the top 20 differentially upregulated and downregulated genes between young and old HSCs-MPP or LT-HSCs. **(E)** Expression of LT-HSCs module score on young and old LT-HSCs. The module score was computed based on a set of marker genes associated to LT-HSCs properties derived from previous publications. **(F)** Heatmap showing average log2FC of genes related to DNA replication stress between young and old HSPCs or LT-HSCs. Genes found statistically significant in at least one of the two cell populations with log2FC < −0.1 and >0.1 were shown. **(G)** Heatmap showing the signature strength of the six ATAC-seq signatures from NMF analysis of indicated BM HSPCs subpopulations from young (three donors, 21–28 years, *n* = 16 subpopulations) and old (four donors, 52–79 years, *n* = 15 subpopulations). **(H)** Signature strength assessment in CD49f^+^ HSCs from young compared with old donors for six NMF signatures. **(I)** Heatmap of the TF motif enrichment of signature 1 and signature 2 shows three main clusters: enriched in signature 1 only (old CD49f^+^ HSCs motifs), shared motifs and signature 2 only motifs (young CD49f^+^ HSCs motifs). **(J)** Log P value enrichment of indicated motifs enriched in young CD49f^+^ HSCs. Dotted red lines mark −log (0.05), indicating statistical significance. **(K)** Log P value enrichment of indicated motifs enriched in old CD49f+HSCs. Dotted red lines mark −log (0.05), indicating statistical significance. **(L)** DEGs in young and old LT-HSCs at transcriptomic level. * < 0.05; *** < 0.001.

To understand the molecular underpinnings for the distinct differentiation capacity of aged human HSCs relative to young HSCs, we generated a single-cell RNA sequencing (scRNAseq) dataset by integrating previously published scRNAseq data from sorted HSPCs from young and old donors (Ainciburu et al., 2023; Li et al., 2025; Mende et al., 2022; Quaranta et al., 2024; Setty et al., 2019) To avoid batch- and donor-related effect, data were harmonized after integration (Table S3). The final dataset encompassed 134,184 HSPCs (89,884 from 17 young donors, 44,300 from 7 old donors). Unsupervised clustering partitioned the full dataset into 21 clusters. By comparing cluster transcriptional profiles with published reference datasets (Quaranta et al., 2024) and according to the expression of HSPCs marker genes, we identified immature (HSC/MPP, cycling-HSCs, myeloid/lymphoid-CMP, MEP), myeloid (CMP, CMP-GMP; granulocyte progenitors; mono-dendritic progenitors), lymphoid (clusters MLP, PreB, and PreNK), megakaryocytic, erythroid (immature and mature erythroid progenitors), and basophil/eosinophil/mast cell transcriptional clusters (Fig. 4 A). By analyzing the distribution across clusters of young and old HSPCs, we observed that old HSPCs were enriched in clusters with HSCs/MPP and erythroid progenitor transcriptional commitment, while young HSPCs displayed an increased proportion of lymphoid progenitors in line with the immunophenotypic analyses (Fig. 4 B). To focus on LT-HSCs, the HSC/MPP cluster was further partitioned into four subclusters, and differential expression (DE) analysis among them allowed the identification of LT-HSCs, MPP, Myelo/lympho-MPP, and Myelo/Ery-MPP (Fig. 4 C). Of note, the composition of the HSC/MPP cluster was similar between young and old subject, with a comparable proportion of cells belonging to LT-HSC cluster, in line with the stable frequencies of CD49f^+^ cells across aging measured through immunophenotypic analyses (Fig. S3 I). DE analyses between young and old HSC/MPP and LT-HSCs revealed increased expression of genes associated to activation (*CD69* and *RGS2*), inflammation (*NR4A1*, *NR4A2*, and *IL1B*), and response to stress and DNA damage (*TIPIN*, *DDIT4*, and *HSPB1*) in old HSPCs subsets, while young HSC showed higher expression of genes involved into metabolism (*RNASEK* and *ALDOA*), differentiation (*CEBP*), cell cycle (*EIF4A1*), and self-renewal (*MAFF* and *AVP*) (Fig. 4 D). Consistently, LT-HSCs from young donors display increased expression of gene modules associated to stemness (Belluschi et al., 2018; García-Prat et al., 2021; Johnson et al., 2024; Kaufmann et al., 2021; Komic et al., 2025; Laurenti et al., 2015; Schönberger et al., 2022; Zeng et al., 2023a, *Preprint*; Zhang et al., 2022) (Fig. 4 E). Moreover, we found deregulated expression of genes involved in DNA replication stress response, fork stability, and replication origin control in aged HSPCs and LT-HSCs (della Volpe et al., 2024; Jacobs et al., 2022), potentially contributing to the observed accumulation of DNA damage in aged cells retrieved upon secondary transplantation (Fig. 4 F).

We then interrogated the chromatin accessibility landscape of HSPCs subpopulations isolated from young and old subjects by assay for transposase accessible chromatin-sequencing (ATAC-seq). We found six signatures by non-negative matrix factorization (NMF) within 31 HSPCs samples, belonging to the distinct HSPCs subpopulations (young: three donors, 21–28 years, *n* = 16; old: four donors, 52–79 years, *n* = 15; Table S4 and Fig. 4 G). When we assessed the strength of all six signatures in young and old CD49f^+^ primitive HSCs samples, signature 1 was significantly enriched in old HSCs, while signature 2 was enriched in young HSCs (Fig. 4 H). Transcription factor (TF) motif analysis within these two signatures yielded three clusters: shared motifs, motifs enriched in young CD49^+^ primitive-HSCs, and motifs enriched in old CD49^+^ primitive-HSCs (Fig. 4 I and Table S5 with enrichment data for all motifs identified). Among the shared motifs are well-known HSCs regulators, including ERG, FLI1, and ETV2 (Kruse et al., 2009; Pimanda et al., 2007; Xu et al., 2017). Importantly, motifs that are accessible in young CD49^+^ primitive-HSCs are associated with cell cycle regulation (E2F) (Iaquinta and Lees, 2007) and hematopoietic lineage decisions (BCL11a and PU.1) (Luc et al., 2016) (Fig. 4 J). In contrast, inflammation response motifs (NFKB and AP1) were enriched in old CD49^+^ primitive-HSCs (Fig. 4 K), in line with transcriptomic data. Additionally, we found that accessibility of HOXB4 motifs, a factor shown to potently expand HSCs (Amsellem et al., 2003; Sauvageau et al., 1995), was enriched in old HSCs (Fig. 4 K). Strikingly, genes involved in these pathways were also found differentially expressed in young and old LT-HSCs at transcriptomic level (Fig. 4 L).

Together, our data suggest that despite a similar repopulating capacity in xenotransplantation experiments, aged BM-derived HSCs show reduced differentiation proficiency with chromatin and transcriptomic alterations in cell cycle, inflammation, and self-renewal factors compared with younger counterparts and prompted us to investigate the biological and functional response of aged HSPCs to activation and proliferative stimuli.

### Human-aged HSPCs show altered proliferation kinetics and impaired clonogenic efficiency upon *ex vivo* activation

**Figure 5.**
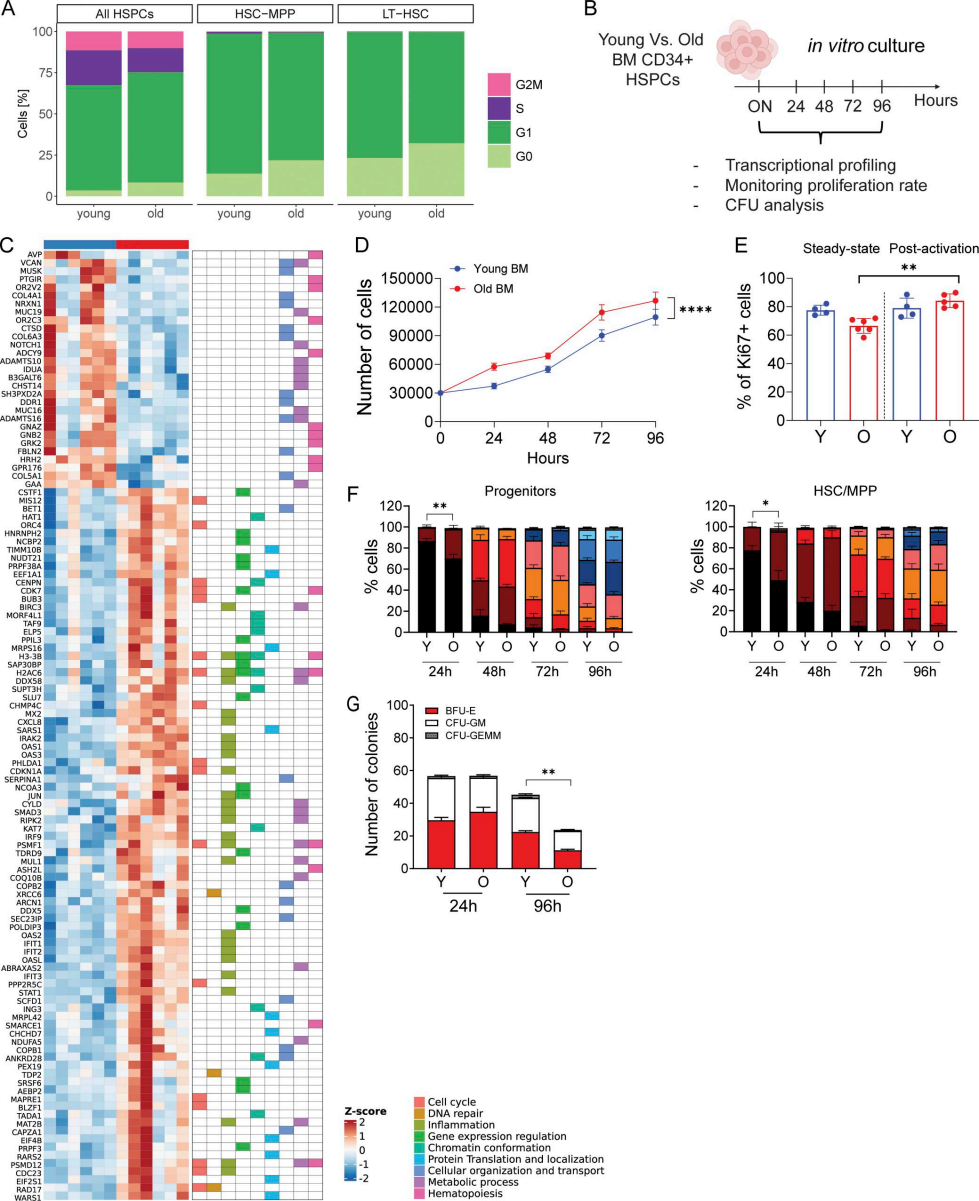
***In vitro* functional characterization of human-aged HSPCs. (A)** Histograms representing the percentage of cells in G0, G1, S, and G2M cell cycle phases for total HSPCs, HSCs-MPP, and LT-HSCs in old and young donors from scRNAseq dataset. **(B)** Experimental scheme for *in vitro* proliferation assay. 3 × 10^5^ BM-derived CD34^+^ cells from young and old individuals were cultured in the presence of the human recombinant cytokines (hSCF 300 ng/ml, hFlt3-L 300 ng/ml, hTPO 100 ng/ml, and hIL-3 60 ng/ml) and analyzed at the indicated time points for transcriptome, proliferation rate, and clonogenic potential. **(C)** Graph showing, on the left, a heatmap with DEGs between young (*n* = 6) and old (*n* = 6) CD34^+^ cells upon overnight activation in culture and, on the right, the Reactome categories associated with the distinct genes. **(D)** Growth curve showing the proliferation of both BM-derived young (*n* = 5) and old (*n* = 5) CD34^+^ cells. Data are shown as mean ± SD. *n* = 5 independent experiments. Statistical test for growth curves: LME model; Y vs. O, P < 0.0001. **(E)** Frequency of Ki67^+^ cells of young (*n* = 4) and aged (*n* = 5) BM-derived CD34^+^ cells at steady-state and at 24 h after culture. Data are shown as mean ± SD. Statistical test: Mann–Whitney; O steady state vs. after activation, P = 0.0015. **(F)** Stacked bar graphs showing the frequencies of cells that completed one or more cell divisions over time in culture in young and old progenitors (left) and HSCs/MPP (right) subsets. *n* = 3 independent experiments Statistical test: Mann–Whitney; progenitors: Y vs. O, P = 0.0019. HSCs/MPP: Y vs. O, P = 0.0165. **(G)** Number of erythroid (BFU-E), myeloid (CFU-GEM), and mixed (CFU-GEMM) colonies generated starting from 800 alive BM-derived CD34^+^ cells of young (*n* = 5) and old (*n* = 5) donors upon activation in culture. Colonies were counted 14 days after plating in CFU-C assay. Data are shown as mean ± SEM. Statistical test: Mann–Whitney; 96 h Y vs. O, P = 0.0079. * < 0.05; ** < 0.01; **** < 0.0001.

**Figure S4.**
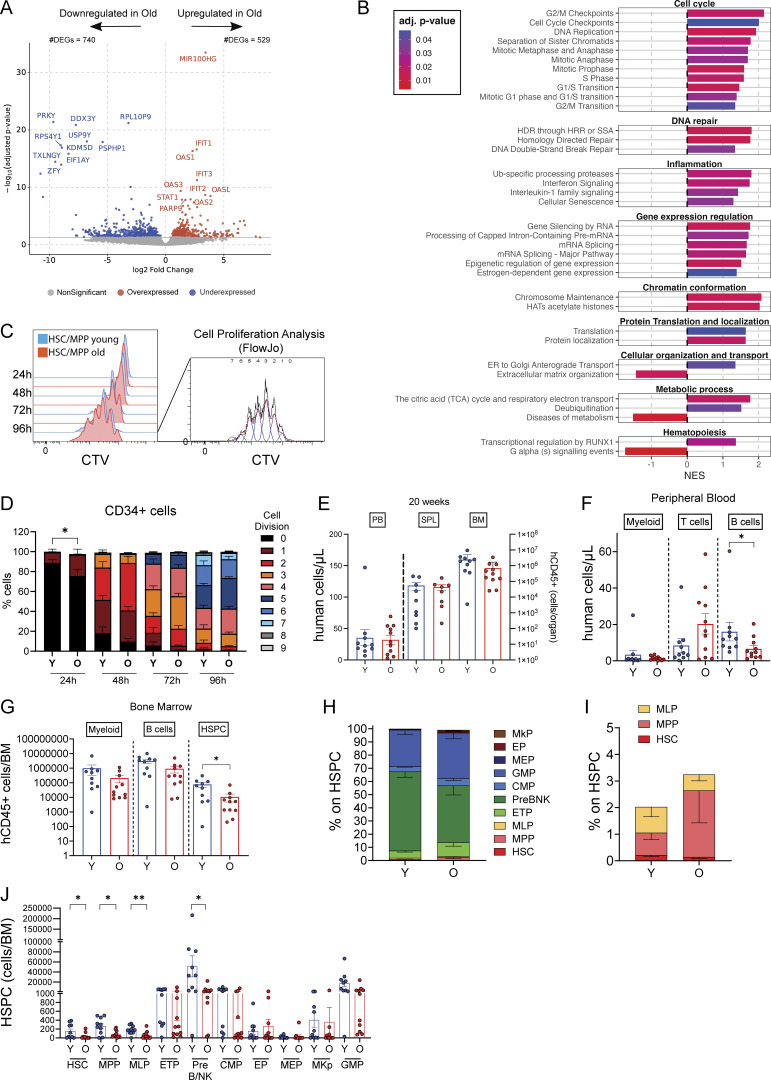
**Transcriptional changes, faster exit from quiescence and reduced repopulation capacity of aged pre-activated HSPCs.** **(A)** Volcano plot showing significant (FDR < 0.05) downregulated and upregulated genes between old and young dataset after an overnight activation in culture. **(B)** Bar plot displaying significant (FDR < 0.05) GSEA categories from the Reactome Database used for the generation of the heatmap shown in Fig. 5 C. Bar length represents the normalized enrichment score (NES) for each category, and the bar color shows the adjusted P-value. **(C)** Left: Representative overlaid histogram in of the dilution of CellTracker dye during proliferation of young and aged sorted HSCs+MPP populations at different time points. Right: Automatic cell proliferation analyses performed by FlowJo software. **(D)** Stacked bar graphs showing the frequencies of cells that completed one or more cell division over time in culture in young (Y) and old (O) total CD34^+^ cells. Data are shown as mean ± SEM. Statistical test: Mann–Whitney; 24 h Y vs. O, P = 0.0378. **(E)** Absolute quantification of human CD45^+^ cells in PB (plotted on the left axis), Spleen, and BM (plotted on the right axis) at 20 wk after transplantation in mice transplanted with young and aged HSPCs. Statistical test: Mann–Whitney; BM Y vs. O, P = 0.051). **(F and G)** Absolute quantification of human subpopulations in PB and BM of mice transplanted with young or aged HSPCs at 20 wk after transplantation. Statistical test: Mann–Whitney; B cells PB Y vs. O, P = 0.0195; HSPCs BM Y vs. O, P = 0.0357. **(H)** Composition of human BM HSPCs compartment in mice transplanted with young or aged HSPCs at 20 wk after transplantation. **(I)** Percentage of human LIN^−^CD3^+^CD38^−^ subpopulations within HSPCs compartment in the BM of mice transplanted with young or aged HSPCs at 20 wk after transplantation. **(J)** Absolute quantification of human HSPCs subpopulations in the BM of mice transplanted with young or aged HSPCs at euthanasia. Statistical test: Mann–Whitney; HSCs, P = 0.0348; MPP, P = 0.0295; MLP, P = 0.0046; PreBNK, P = 0.017. In all the graphs, data are shown as mean ± SEM. * < 0.05; ** < 0.01.

We first exploited the generated scRNAseq data to infer the cell cycle state of HSPCs, HSCs/MPP, and LT-HSCs from young and old donors at steady state (Fig. 5 A), based on marker genes associated to the distinct cell cycle phases (see also Materials and methods section) (Quaranta et al., 2024). This analysis revealed that a small fraction (5–10%) of cells within HSPCs display transcriptional signature associated to G0 phase, which increases moving from HSPCs→ HSC/MPP→ LT-HSCs, validating our G0 classification. Moreover, old HSPCs, HSC/MPP, and LT-HSCs were found more quiescent with respect to their young counterparts (Fig. 5 A). To investigate the responses of young and old HSPCs to activation, we stimulated BM CD34^+^ cells isolated from young (<35 years old, *n* = 6) and old (>60 years old, *n* = 6) subjects with a cocktail of human recombinant cytokines and analyzed their transcriptional profiles, proliferation rates, and clonogenic potential (Fig. 5 B). Global gene expression analyses identified 1,269 statistically significant differentially expressed genes (DEGs) (false discovery rate [FDR] < 0.05) in CD34^+^ cells from old subjects compared with their younger counterparts after an overnight activation, of which 529 were upregulated and 740 were downregulated (Fig. S4 A and Table S6). Gene set enrichment analysis (GSEA) against hallmark gene sets from the Reactome Database revealed upregulation of genes associated with cell cycle (*RAD17*, *CDK7*, *BUB3*, and *CENPN*), inflammation (*IFIT1-3*, *STAT1*, *OAS1-,3*, *SMAD3*, *BIRC3*, and *CDC23*), chromatin conformation and gene expression regulation (*H3-3B*, *TAF9*, *HAT1*, *ELP5*, *KAT7*, *SLU7*, *H2AC6*, and *JUN*), and protein translation and localization (*RARS2*, *EIF4B*, *WARS1*, *PEX19*, *SARS1*, *TIMM10B*, and *MRPS16*) categories in activated old CD34^+^ cells with concomitant downregulation of genes belonging to hematopoiesis (*AVP*, *PTGIR*, *OR2V2*, *ADCY9*, *GNAZ*, *GNB2*, *GRK2*, *HRH2*, *GPR176*, and *ASH2L*) and cellular organization and transport categories (*SERPINA1*, *COPB2*, *ARCN1*, *SEC23IP*, *SCFD1*, *COPB1*, and *CAPZA1*) (Fig. 5 C and Fig. S4 B).

Growth curve analysis showed that aged HSPCs displayed a higher proliferation rate, mainly at early time points after activation. Moreover, in line with the cell cycle state measured in our scRNAseq, old HSPCs display reduced ki67^+^ cells before culture, but higher percentage of proliferating cells after activation as compared with younger counterparts (Fig. 5, D and E), suggesting a faster exit from quiescence. We next analyzed cell division kinetics on sorted HSC/MPP (defined as LIN^−^CD34^+^CD38^−^CD45RA^−^ cells) and more committed subsets, including myeloid, erythroid, and megakaryocytes progenitors (defined as LIN^+^CD34^+^CD38^+^CD10^−^CD7^−^ cells) (Fig. 5 F and Fig. S4, C and D). We found that a higher percentage of total CD34^+^ cells as well as of HSC/MPP and committed subsets isolated from old donors completed one or more cell divisions at earlier time points in comparison to younger counterparts. These early differences in proliferation rate diminished over time, with a similar profile of cell divisions observed at later time points between young and aged HSPC subsets (Fig. 5 F). By performing clonogenic assay on pre-activated young and aged CD34^+^ cells, we found a comparable number of colonies early upon activation followed by a significant reduction in aged HSPCs–derived colonies at later time points (Fig. 5 G).

Taken together, these data suggest that, after *ex vivo* activation, aged HSPCs showed a faster exit from quiescence, resulting in altered proliferation rates, characterized by transcriptional changes in cell cycle–related and inflammatory gene categories that may ultimately contribute to reduced clonogenicity upon prolonged time in culture.

### Activated aged HSPCs retain differentiation potential but display impaired long-term fitness after *in vivo* transplantation

**Figure 6.**
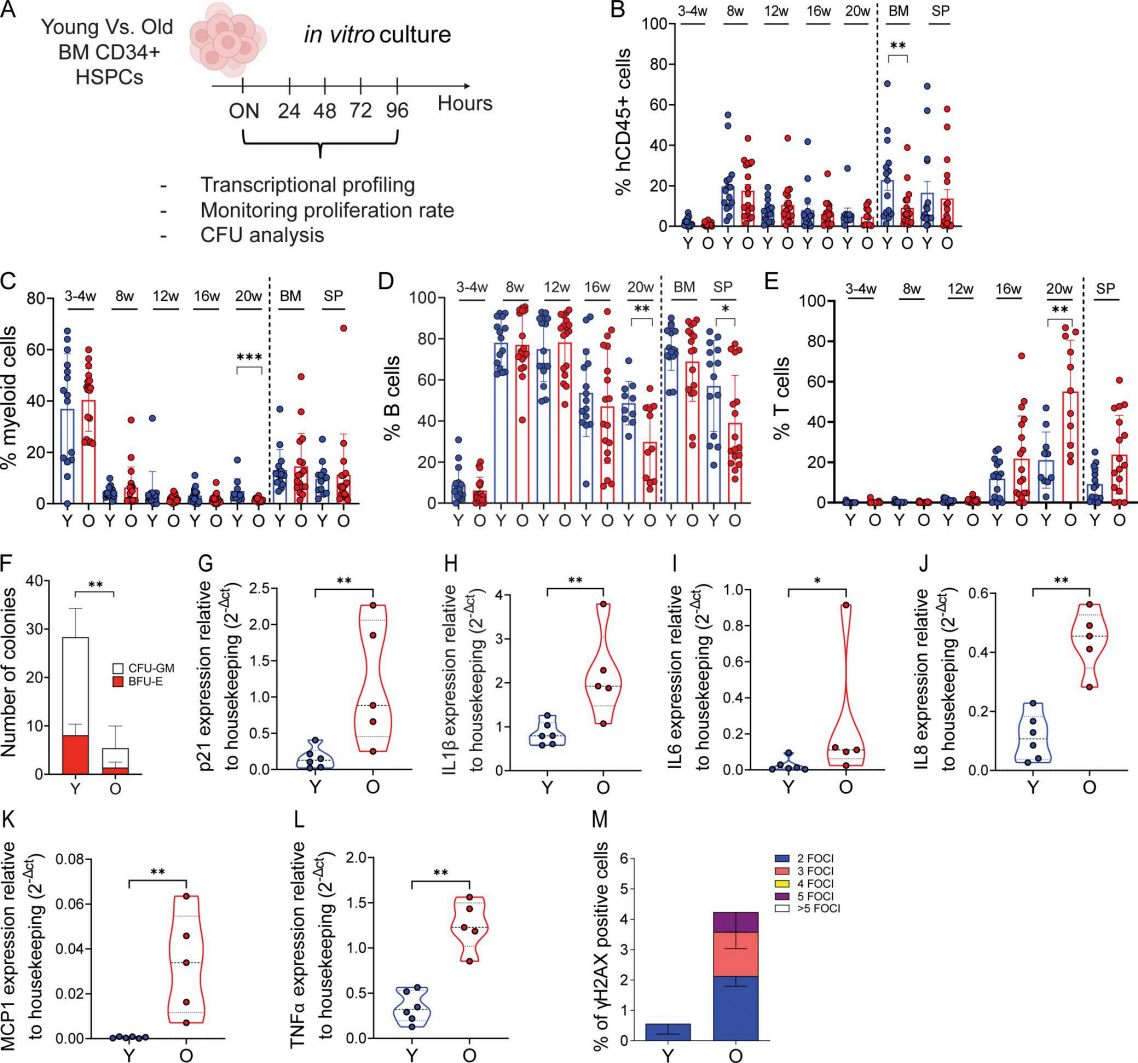
***In vivo* functional characterization of pre-activated human-aged HSPCs. (A)** Experimental scheme for xenotransplantation experiment where human CD34^+^ cells from young (*n* = 4) and old donors (*n* = 4) were pre-cultured overnight prior transplantation into NSG (NOD.Cg *Prkdc*scid*Il2rg*tm1Wjl/SzJ) mice (*n* = 3 mice for each donor). **(B)** Percentage of human engrafted cells (CD45^+^ cells) in PB over time as well as in BM and spleen (SP) at euthanasia in mice transplanted with young or aged HSPCs. Statistical test: Mann–Whitney; BM Y vs. O, P = 0.0099. **(C–E)** Percentage of human myeloid cells (CD13^+^ cells [C]), B cells (CD19^+^ cells [D]), and T cells (CD3^+^ cells [E]) in PB over time as well as BM and SP at 20 wk in mice transplanted with young or old HSPCs. Statistical test: Mann–Whitney; PB myeloid cells 20 wk Y vs. O, P = 0.0008; PB B cells 20 wk Y vs. O, P = 0.0215; Spleen B cells Y vs. O, P = 0.0149. PB T cells 20 wk Y vs. O, P = 0.0018. **(F)** Number of colonies generated starting from the same amount of CD34^+^ cells isolated from transplanted mice at 20 wk. Statistical test: Mann–Whitney; Y vs. O, P = 0.0022. **(G–L)** Relative mRNA expression of cell cycle inhibitor *p21* and inflammatory genes *IL1β*, *IL6*, *IL8*, *MCP1*, and *TNFα* in human CD34^+^ cells isolated from BM of mice transplanted with young and aged HSPCs (20 wk). Statistical test: Mann–Whitney; *p21* Y vs. O, P = 0.0087; *IL1β* Y vs. O, P = 0.0087; *IL6* Y vs. O, P = 0.0173; *IL8* Y vs. O, P = 0.0043; *MCP1* Y vs. O, P = 0.0043; *TNFα* Y vs. O, P = 0.0043. Gene expression was measured by quantitative real-time PCR and represented as 2^−ΔCt^ relative to housekeeping. **(M)** Frequencies of cells showing nuclear γH2AX foci in human CD34^+^ cells isolated from BM of mice transplanted with young and old HSPCs at 20 wk. Statistical test: Mann–Whitney test. * < 0.05 ** < 0.01; *** < 0.001.

We next transplanted pre-activated CD34^+^ cells derived from young (*n* = 4) and old (*n* = 4) donors into NSG mice to assess their *in vivo* hematopoietic output (Fig. 6 A). We found no significant differences in the kinetics and the frequency of human CD45^+^ hematopoietic cells in PB (Fig. 6 B), while we observed a reduced human graft in the BM of mice transplanted with old CD34^+^ cells at 20 wk after transplant (Fig. 6 B and Fig. S4 E). Of note, mice transplanted with old CD34^+^ cells showed a statistically significant reduction in both myeloid and B cell output accompanied by a relative increase of T cell content in the PB at the time of euthanasia (Fig. 6, C–E). These changes in the lymphoid compartment were also observed in the spleen of the transplanted mice (Fig. 6, D and E). We confirmed by quantitative analyses a reduced B cell output both in PB and in BM from mice belonging to the old group accompanied by a reduction of the overall HSPCs content at 20 wk (Fig. S4, F and G). We also found an increased frequency of LIN^−^CD34^+^CD38^−^ cells and a reduced lymphoid progenitor contribution in the human HSPCs engrafted in the BM of the mice transplanted with old CD34^+^ cells (Fig. S4, H and I). In contrast, absolute quantification of distinct HSPC subsets in the BM of transplanted mice unveiled a reduced amount of primitive but not of more committed HSPCs progenitors in the old group (Fig. S4 J). Moreover, CD34^+^ cells retrieved at the endpoint from mice transplanted with old CD34^+^ cells showed reduced clonogenic potential (Fig. 6 F) and increased gene expression of cell cycle inhibitor *p21* and several pro-inflammatory cytokines (including *IL6*, *IL8*, *IL1β*, *MCP1*, and *TNFα*) compared with younger counterparts (Fig. 6, G–L), together with the accumulation of DNA double-strand breaks, detected through the phosphorylation of histone H2A.X (γH2AX) (Fig. 6 M).

These data suggest that, although pre-activated aged HSPCs reconstitute all main blood cell lineages (myeloid, T, and B cells) shortly upon transplantation, they are likely unable to counteract transplant-related proliferation stress, resulting in detrimental cellular responses that may impair their functionality in the long term.

### Establishing an *in vivo* model of proliferation stress by low cell dose transplantation

**Figure 7.**
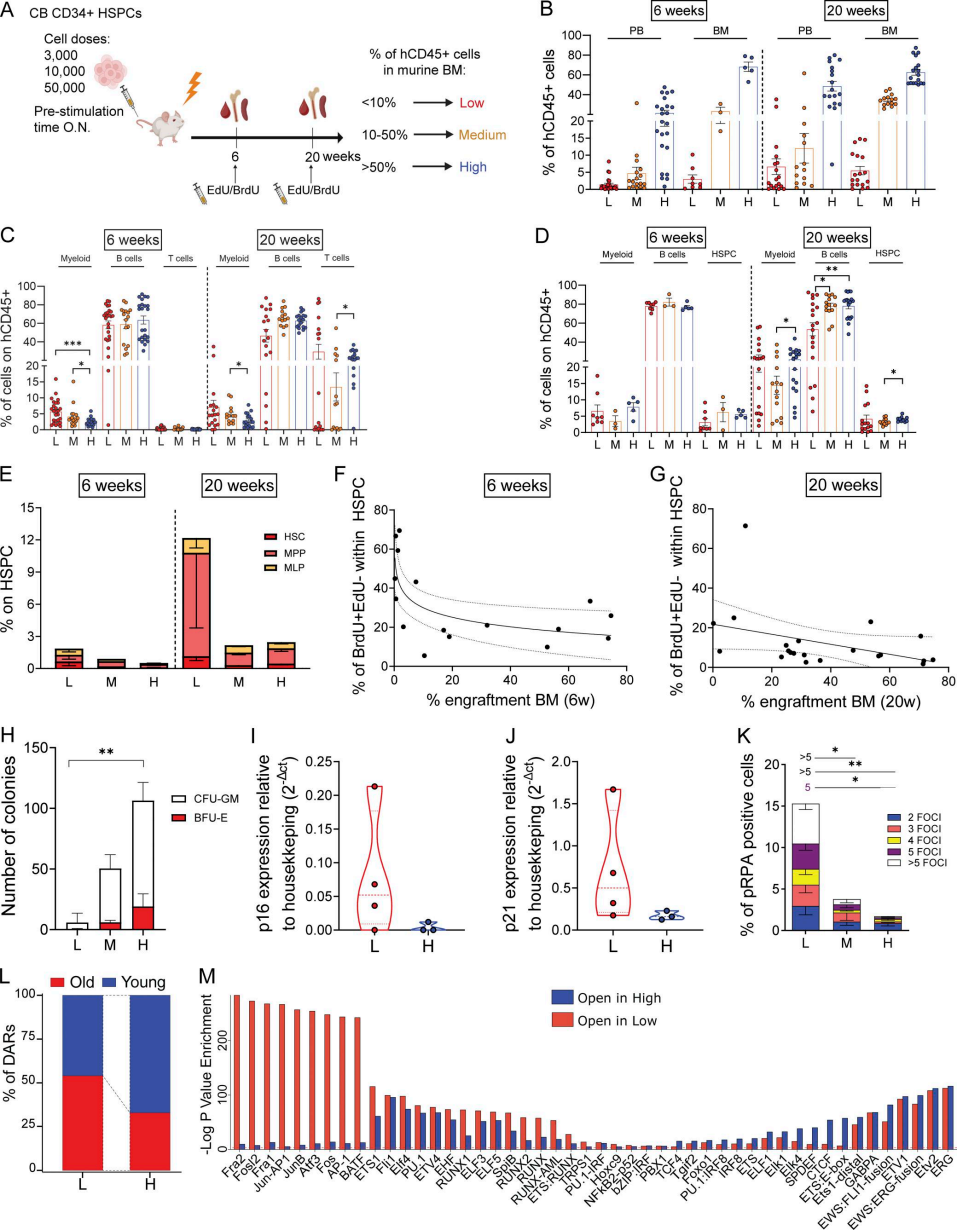
***In vivo* modeling of HSPCs proliferation stress. (A)** Schematic representation of the *in vivo* transplantation setting. Three groups of NSG (NOD.Cg *Prkdc*scid*Il2rg*tm1Wjl/SzJ) mice were transplanted with different doses of CB-derived CD34^+^ cells (*n* = 3 independent experiments; *n* = 35 mice for each experiment). After evaluation of human cell content in the murine BM at euthanasia, mice were divided into three distinct categories according to the level of engraftment: Low-engrafted (L, <10% hCD45^+^ cells), middle-engrafted (M, 10–50% of hCD45^+^ cells), and high-engrafted (H, >50% hCD45^+^ cells). Mice with engraftment below 0.05% in the murine BM were considered not engrafted and excluded from the graphical representation. **(B)** Percentage of human engrafted cells (CD45^+^ cells) in PB and BM of transplanted mice at both 6 and 20 wk after transplantation. **(C)** Percentage of human myeloid cells (CD33^+^CD66b^+^ cells), B cells (CD19^+^ cells), and T cells (CD3^+^ cells) in PB of transplanted mice at both 6 and 20 wk after transplantation. Statistical test: Mann–Whitney; 6 wk myeloid: L vs. H, P = 0.0004; M vs. H, P = 0.022. 20 wk myeloid: M vs. H, P = 0.049; T cells: M vs. H, P = 0.036. **(D)** Percentage of human myeloid cells (CD33^+^CD66b^+^ cells), B cells (CD19^+^ cells), and HSPCs (CD34^+^ cells) in BM of transplanted mice at both 6 and 20 wk after transplantation. Statistical test: Mann–Whitney; 20 wk myeloid: M vs. H, P = 0.0049; B cells: L vs. M, P = 0.021; L vs. H, P = 0.0082; HSPCs: M vs. H, P = 0.049) **(E)** Percentage of primitive HSPCs subsets within BM HSPCs compartment in transplanted mice at both 6 and 20 wk after transplantation. **(F and G)** Correlation between the percentage of HSPCs that entered S phase per hour (BrdU^+^EdU^−^) with human engraftment in murine BM at 6 and 20 wk after transplantation. Statistical test: Spearman r; 6 wk, P = 0.0147; 20 wk, P = 0.014. **(H)** Number of colonies generated starting from the same amount of CD34^+^ cells isolated from transplanted mice at 20 wk. Statistical test: Mann–Whitney; L vs. H, P = 0.0046. **(I and J)** Relative mRNA expression of cell cycle inhibitors *p16* and *p21* in human CD34^+^ cells isolated from low or high-engrafted mice (20 wk). **(K)** Frequencies of cells showing nuclear pRPA foci in human CD34^+^ cells isolated from BM of transplanted mice (20 wk). Only statistically significant P values were reported. Statistical test: Mann–Whitney; >5 foci L vs. M, P = 0.041; L vs. H, P = 0.009. 5 foci L vs. H, P = 0.043. **(L)** DARs detected in human CD34^+^ isolated from mice transplanted with the low and high cell input of CB-derived HSPCs from ATAC-seq analyses, annotated with unique old and young ATAC signatures on sorted primitive HSPCs (as defined in Fig. 4, G–I). **(M)** List of shared motifs associated to transcription-binding sites enriched in high-engrafted (blue) and low-engrafted (red) mice. Red dashed line indicates adjusted P value < 0.05. Statistical test for association of DARs to young and old signature: Chi-square test; L vs. H. P = 4.3e−113. * < 0.05; ** < 0.01; *** < 0.001.

To study the impact of proliferative stress on hematopoietic reconstitution *in vivo*, we hypothesized that, when transplanted in limited numbers, HSPCs may undergo an increased number of cell divisions to repopulate the BM than when transplanted in higher amounts (Laurenti et al., 2015). We therefore transplanted overnight cultured neonatal CB-derived HSPCs at different cell doses (50,000, 10,000 and 3,000) in sublethally irradiated immune-compromised mice and tested their repopulating capacity and blood lineage reconstitution at early (6 wk) and late (20 wk) phases after transplantation (Fig. 7 A). Concomitantly, we combined cell number measurements with a previously established short timescale dual pulse labeling method (Akinduro et al., 2018) that allows us to quantify the number of cells entering S-phase per hour. Specifically, before euthanasia, NSG mice were subjected to an initial intravenous EdU pulse (2 h) followed by a second intravenous injection of BrdU. By measuring the cells positive for one or both markers, we estimated the number of cells in active proliferation at early and late phases after transplantation.

**Figure S5.**
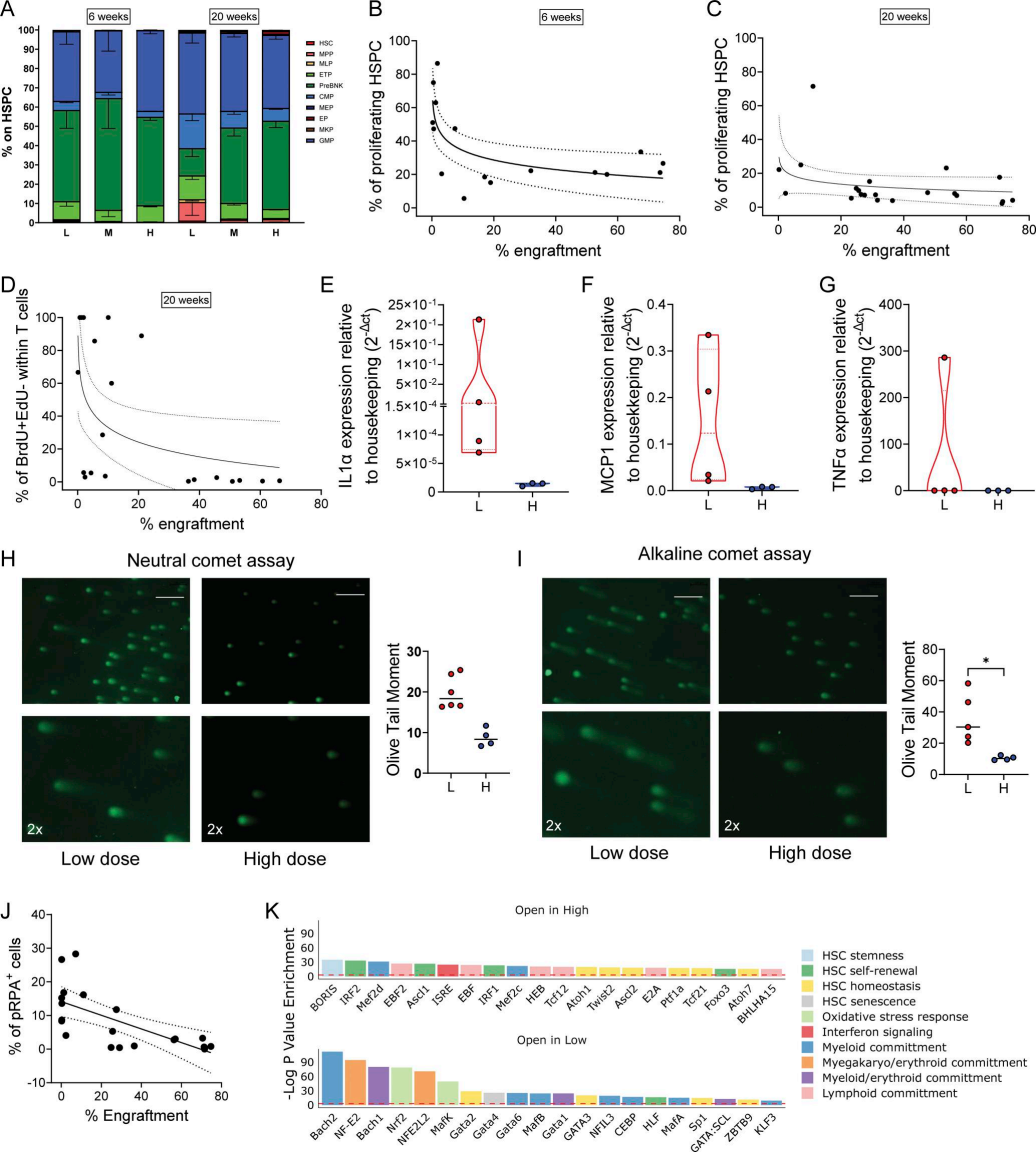
**Proliferative dynamics, DNA damage and transcriptional programs of human HSPCs in low-cell dose transplantation assay.** **(A)** Composition of human BM HSPCs compartment in low-, middle-, and high-engrafted mice at both 6 and 20 wk after transplantation. **(B and C)** Correlation between the percentage of total proliferating HSPCs (BrdU^+^EdU^−^; BrdU^+^EdU^+^; BrdU^−^EdU^+^) with human engraftment in murine BM at 6 and 20 wk after transplantation. Statistical test: Spearman r; 6 wk, P = 0.041; 20 wk, P = 0.0115. **(D)** Correlation between the percentage of T cells that entered S phase per hour (BrdU^+^EdU^−^) with human engraftment in PB at 20 wk after transplantation. Statistical test: Spearman r; P = 0.0002. **(E–G)** Relative mRNA expression of inflammatory genes *IL1α*, *MCP1*, and *TNFα* in human CD34^+^ cells isolated from low- and high-engrafted mice at 20 wk. Statistical test: Mann–Whitney; *IL1α,* P = 0.051; *MCP1*, P = 0.0571; and *TNFα*, P = 0.0571. **(H and I)** Representative images and Olive Tail Moment average values of neutral (H) or alkaline (I) comet assays conducted on CD34^+^ isolated from low- or high-engrafted mice at 20 wk after transplantation. White lines indicate the length of 100 µm. Statistical test: Mann–Whitney; alkaline: L vs. H, P = 0.0159. **(J)** Correlation between the frequency of pRPA^+^ cells within HSPCs with human engraftment in murine BM at 20 wk after transplantation. Statistical test: Spearman r; P = 0.0003. **(K)** List of top 20 TFs associated to enriched binding sites unique for high-engrafted (open in high) and low-engrafted (open in low) mice. Each TF is colored according to its biological function in HSCs. * < 0.05.

To assess the effect of proliferation stress on the engraftment, we further stratified our transplanted mice according to their engraftment in the BM (Fig. 7 B; low-engrafted: <10% of hCD45^+^ cells; middle-engrafted: 10–50% of hCD45^+^ cells; high-engrafted: >50% of hCD45^+^ cells). Interestingly, we found that low-engrafted mice displayed a phenotype of hematopoietic reconstitution like that observed after transplantation of old CD34^+^ cells in NSG mice. Indeed, we found slightly increased myeloid output at 6 wk in the PB of low-engrafted mice and reduced B cell production with a tendency of increased T cell output (four out of nine mice) in the PB at 20 wk (Fig. 7 C). At the same time point, low-engrafted mice showed a significant decrease in the percentage of human HSPCs in the BM, with an expansion of more primitive cells (LIN^−^CD34^+^CD38^−^ cells) and a concomitant decrease in the percentage of B lymphoid progenitors (PreB/NK) (Fig. 7, D and E; and Fig. S5 A). Moreover, the evaluation of EdU and BrdU uptake revealed a statistically significant negative correlation between the percentage of HSPCs entering in S phase per hour (defined as EdU^−^BrdU^+^ cells) (Akinduro et al., 2018) or total proliferating HSPCs (EdU^−^BrdU^+^, EdU^+^BrdU^+^, and EdU^+^BrdU^−^ cells) and the human graft in the BM at 6 and 20 wk after transplantation (Fig. 7, F and G; and Fig. S5, B and C). In addition, we also observed a higher T cell proliferation in low-engrafting mice, suggesting that the increased T cell frequencies observed at 20 wk were likely due to mature T cell proliferation in the PB rather than *de novo* T cell production (Fig. 5 D).

Strikingly, when plated at the same number, we found a reduced clonogenic capacity of CD34^+^ cells retrieved at the time of euthanasia (20 wk after transplantation) from the BM of low-engrafted mice with respect to middle/high-engrafted mice (Fig. 7 H). Moreover, CD34^+^ cells isolated from mice transplanted with low cell doses displayed a significant increase in expression levels of senescence-associated cell cycle inhibitors *p16* and *p21* (Fig. 7, I and J) as well as in inflammatory genes *IL1-α*, *MCP-1*, and *TNF-α* (Fig. S5, E–G). The proliferative stress imposed by the low cell dose was associated also with the higher presence of both single- and double-stranded DNA breaks (Fig. S5, H and I). Along the same line, CD34^+^ cells isolated from low-engrafting mice showed a statistically significant increased frequency of pRPA-positive cells (Fig. 6 K), a well-established marker of DNA replicative stress (Ciccia and Elledge, 2010), which inversely correlated with the engraftment levels in the BM (Fig. S5 J). Next, we profiled the chromatin state of human CD34^+^ cells retrieved from high- and low-engrafted mice by ATAC-seq, and we evaluated differentially accessible regions (DARs) belonging to the signature 1 (enriched in old HSCs) and 2 (enriched in young HSCs) from the ATAC-seq analyses shown in Fig. 4. We found an enrichment of DARs belonging mainly to the regions associated with the “Young” signature (3,679 versus 1,806) in the high-engrafted mice, while the low-engrafted mice display enrichment of regions associated to the “Old” signature (2,697 versus 3,167) (Fig. 7 L). Analyses of shared TF-binding site motifs between high- and low-engrafted mice revealed differential enrichment of AP-1 and RUNX1 motifs in low-engrafted mice, while high-engrafted mice showed enrichment of ETS/PU.1 motifs, consistently with the old and young signature from the ATAC-seq data on human HSPC subpopulations (Fig. 7 M). These analyses imply that our low cell input model recapitulates the chromatin state of aged HSPCs. Finally, by analyzing the top 20 unique TF motifs for low- and high-engrafted mice, we found that HSPCs from low-engrafted mice display enrichment in TF-binding site associated to myeloid/erythroid specification and oxidative stress, while TF-binding site motifs from mice transplanted with high cell dose are mainly associated to lymphoid differentiation and HSCs self-renewal (Fig. S5 K).

Taken together, these findings indicate that the proliferative burst imposed by low cell transplantation in human HSPCs recapitulates age-associated phenotypic, functional, and chromatin changes of human hematopoiesis and leads to the activation of detrimental cellular responses characterized by DNA damage accumulation and inflammatory programs, ultimately leading to altered hematopoietic output and premature exhaustion of the HSPCs compartment.

## Discussion

Here, we provide a comprehensive quantitative characterization of the phenotypic and molecular changes occurring during hematopoietic aging in a cohort of 73 healthy donors, comprising PB and BM collected from pediatric as well as young, middle-aged, and old subjects. By virtue of quantitative measurements of the overall hematopoietic system, we found that human hematopoiesis in the elderly at steady state is characterized by an overall reduction of differentiated cell production, primarily impacting lymphoid and erythroid lineages, accompanied by a generally stable myeloid output with a significant decrease in DC populations. Thus, we conclude that the reported myeloid skewing observed in previous works (Pang et al., 2011) may primarily result from a reduction in lymphoid and erythroid lineages, rather than from an actual enhancement in myeloid output, despite having a comparable number of human HSCs and MPPs throughout life. Interestingly, the immunophenotypic profiles in our limited sample cohort did not appear to be influenced by the CH mutations identified through exome sequencing. These mutations were infrequent and exhibited low VAFs, suggesting a minimal impact on the observed cellular phenotypes. However, increasing the sample size may reveal subtle differences that were not detectable in this initial analysis.

From a functional standpoint, although LT-HSCs from aged and young donors showed similar behavior when analyzed as a pool, single-cell assay revealed that LT-HSCs from aged donors exhibited a marked reduction in differentiation efficiency and a pronounced skewing toward erythroid and myeloid outputs compared with their younger counterparts. These functional alterations were in line with the transcriptional changes detected through scRNAseq analysis, including decreased expression of key genes involved in stem cell self-renewal, maintenance of stemness, and balanced myeloid-lymphoid differentiation. These findings point to a progressive erosion of transcriptional programs that sustain multipotency and long-term self-renewal in LT-HSCs with age. Interestingly, when transplanted as a pool of CD34^+^ cells, aged and young LT-HSCs exhibited comparable repopulating frequencies and lineage output *in vivo*, even upon secondary transplantation, extending previous findings that showed similarities between young and old HSCs in xenotransplants up to 12 wk (Amoah et al., 2022; Kuranda et al., 2011). The differential outcomes between *in vitro* single-cell differentiation assays and xenotransplantation experiments might result from: (1) inherent limitations of NSG xenotransplantation models to faithfully capture subtle age-related changes in human HSC differentiation potential, especially toward the myeloid, erythroid, and megakaryocyte lineages; (2) a bystander effect of supportive MPP and progenitor cells present in bulk CD34^+^ cells that might have contributed to the observed hematopoietic reconstitution upon transplantation. At this stage, we cannot rule out the possibility that by employing alternative immunodeficient mouse models to better support individual human LT-HSC engraftment studies (Dykstra et al., 2007; Notta et al., 2011) might allow deeper capture of variations in repopulating potential and lineage-specific variations of aged LT-HSCs.

Interestingly, we found that LT-HSCs from aged donors retrieved from recipient animals showed increased levels of DNA damage, aligning with the deregulation of DNA replication stress response and LT-HSC activation genes observed in our scRNAseq datasets. These data imply that while aged HSPCs can still functionally repopulate the host, they may do so under a state of elevated cellular stress and risk for genomic instability. In addition, we cannot exclude that the clonal architecture of reconstituted hematopoiesis differs significantly between young and aged donors. Specifically, grafts derived from aged LT-HSCs may exhibit reduced clonal diversity or increased clonal dominance, as recently demonstrated in human transplantation studies (Spencer Chapman et al., 2024), potentially reflecting a diminished pool of truly multipotent stem cells with aging. Future clonal tracking studies will provide definitive answers on the clonal output of transplanted aged HSPCs.

Using a novel inflammation-recovery xenotransplantation model, some of us recently discovered an inflammatory memory in LT-HSC (HSCs-iM) subset that is equivalent to a subset of HSCs found in the BM of old adults (Zeng et al., 2023a, *Preprint*). Accordingly, many of the top motifs enriched in the chromatin accessibility signature from aged *vs.* young immunophenotypic CD49f^+^ primitive HSCs (AP-1 and NFkB) at steady state are the same motifs enriched in HSCs-iM after recovery from inflammatory challenge (Zeng et al., 2023b, *Preprint*). Considering these findings, we hypothesize that repeated stress events in the form of acute infections or inflammatory stimuli across the human lifespan are an “allostatic load” that impacts HSCs function (McEwen, 2003). Moreover, this inflammatory memory of aged HSPCs may sensitize them to damage, heightening their response to the proliferative stress induced by *ex vivo* activation and transplantation, as previously reported for skin epithelial cells and akin to trained immunity in HSCs (Bogeska et al., 2022; Cirovic et al., 2020; de Laval et al., 2020; Kaufmann et al., 2018; Naik et al., 2017; Naik and Fuchs, 2022). Indeed, when activated, aged HSPCs displayed a higher proliferation rate with a more rapid exit from dormancy associated with accumulation of proliferation stress-induced DNA damage, cell cycle inhibitors, inflammatory cytokines, and reduced long-term engraftment, likely due to deregulation of genes involved in DNA replication initiation, fork stability, and DNA damage response checkpoint genes, in line with data in murine old HSCs (Flach et al., 2014). The enhanced ability of aged HSPCs and of the sorted HSCs/MPP subset to respond to mitogenic stimuli *ex vivo* appears in contrast with the recent evidence reporting that individual CD49f^+^ primitive HSCs showed age-associated delayed timing of first division and prolonged G1 phase (Hammond et al., 2023) and likely highlights a differential response to distinct mitogenic stimuli between a pool of progenitors/stem cells compared with highly purified individual primitive cells. Instead, our findings align with literature in murine HSPCs reporting increased DNA replication stress in aged HSCs during the S phase (Flach et al., 2014) and with the evidence that DNA damage in LT-HSCs is a uniform consequence of treating mice with a range of stimuli, including viral infections, that provoke HSCs cycle entry (Jacobs et al., 2022; Walter et al., 2015).

Notably, our novel xenotransplantation-based model with low-input CB-derived HSPCs faithfully recapitulates several features observed in physiologically aged hematopoiesis, including reduced lymphoid output, increased inflammatory cytokine production, and altered response to DNA replication stress. In addition, chromatin accessibility profiling of HSPCs isolated from the low-cell input xenotransplantation model revealed an enrichment of TF-binding motifs, including AP-1—that are also prominently found in LT-HSCs isolated from aged individuals. This shared epigenetic signature suggests that the proliferative stress imposed on HSPCs transplanted at low cell dose may induce a chromatin landscape resembling that of physiologically aged stem cells.

From a preclinical perspective, our *in vivo* proliferation-stress model can also be used to test innovative strategies to mitigate (1) stress-induced defects of aged human hematopoiesis, (2) the reduced HSPCs clonality observed in transplanted patients in the long term (Lahav et al., 2005), and (3) detrimental responses associated with *ex vivo* HSPCs culture and manipulation in autologous gene therapy settings, given that their applications to older individuals for the treatment of cancer and/or degenerative diseases are predicted to increase in the next decades.

Altogether, our data show that human hematopoietic aging is associated with overall hematopoietic reduction with impaired functionality and altered proliferation-stress responses acting at the HSCs level. Moreover, we provide a powerful *in vivo* model for studying aging of human HSPCs with clinically relevant implications, and overcome the limitations imposed by low availability of human-aged BM samples in the context of preclinical research.

## Materials and methods

### Human hematopoietic samples in the Italian cohort

We complied with all the ethical regulations for retrieving biological materials from healthy donors. To characterize the hematopoietic compartment over aging, we collected BM samples from 9 pediatric (0–18 years), 13 young adult (18–40 years), 20 middle-aged (40–60 years), and 31 aged (>65 years) healthy individuals, as well as PB samples from 12 pediatric (0–18 years), 17 young adult (18–30 years), 8 middle-aged (40–60 years), and 19 aged (>65 years) healthy subjects. Pediatric BM samples were collected as residual excess material from subjects undergoing BM harvest as donors for transplantation, after parents’ signed informed consent. Aged BM samples were collected from subjects who underwent hip replacement surgery at San Raffaele Hospital, Milan, after obtaining written informed consent. BM CD34^+^ cells from young healthy donors for functional assays were collected at San Raffaele Hospital or purchased from Lonza. For the phenotypic analyses, the sample size was determined by the number of individuals for whom excess material was available after signing informed consent for research protocols approved by the San Raffaele Scientific Institute’s Ethics Committee (according to protocol TIGET09). We excluded from our analyses all subjects with compromised immune functions and previous history of cancer or cancer treatments. We also excluded subjects that were positive for HBV, HCV, and HIV infections. We included only subjects that scored as “no frail” upon Edmonton Frailty Scale (Perna et al., 2017) evaluation.

### Human hematopoietic samples in the North American cohort

Human BM mononuclear cells were purchased from Lonza, collected from young healthy volunteers from Toronto with informed consent, or collected from patients undergoing hip replacement surgery at the Traumatology and Orthopedics Hospital Lomas Verdes, Mexico, with verbal consent, as determined by the Institutional Ethical Board. The Mexican samples were confirmed to have no dysplasia of any hematopoietic lineages by histological and complete blood count analysis (Aguilar-Navarro et al., 2020). Ethical approval was obtained from the Institutional Review Board (R-2012-785-092). Samples were viably frozen and stored at −150°C. All Toronto hematopoietic samples were collected with informed consent according to the procedures approved by the University Health Network (UHN) Research Ethics Board (REB 01-0573-C). CB samples were obtained from Trillium and Credit Valley Hospital and William Osler Health Centre, processed as previously described, and stored viably as CD34-enriched HSPCs at −150°C (Xie et al., 2019). BM samples were thawed, and CD34 enrichment from BM samples was performed by positive selection with the CD34 Microbead kit (Miltenyi) with MACS magnet technology (Miltenyi) and stained with the following panel for HSPCs immunophenotypic analysis: FITC-anti-CD45RA, PE-anti-CD90, APC-anti-CD10, PE-Cy5-anti-CD49f, BV711-anti-CD19, APC-Cy7-anti-CD34, PE-Cy7-anti-CD38, biotin-anti-CD135, Alexa-Fluor-anti-CD7, V421-anti-CD33, BV650-anti-CD71, streptavidin-Qdot605, and propidium iodide.

### Whole blood dissection staining

PB samples and BM aspirate were stained according to the published whole blood dissection (WBD) protocol (Basso-Ricci et al., 2017). In brief, after RBC lysis, the samples were labeled with the following fluorescent antibodies:

• Mouse anti-human CD3-BV605 (clone: OKT3; 317322; BioLegend); verified reactivity: human; application: flow cytometric analysis of antibody surface-stained cells. Dilution: 1:50.
• Mouse anti-human CD56-PC5 (clone: 5.1H11; 362516; BioLegend); verified reactivity: human; application: flow cytometric analysis of antibody surface-stained cells. Dilution: 1:50.
• Mouse anti-human CD14-BV510 (clone: M5E2; 301842; BioLegend); verified reactivity: human, cynomolgus, and rhesus; application: flow cytometric analysis of antibody surface-stained cells. Dilution: 1:50.
• Mouse anti-human CD33-BB515 (clone: WM53; 564588; BD Biosciences); verified reactivity: human (QC testing); application: flow cytometry (routinely tested). Dilution: 1:50.
• Mouse anti-human CD41/CD61-PC7 (clone: A2A9/6; 359812; BioLegend); verified reactivity: human; application: flow cytometric analysis of antibody surface-stained cells. Dilution: 1:50.
• Mouse anti-human CD66b-BB515 (clone: G10F5; 564679; BD Biosciences); verified reactivity: human (QC testing); application: flow cytometry (routinely tested). Dilution: 1:50.
• Mouse anti-human CD7-BB700 (clone: M-T701; 566488; BD Biosciences); verified reactivity: human (QC testing), rhesus, cynomolgus, and baboon (reported); application: flow cytometry (routinely tested). Dilution: 1:50.
• Mouse anti-human CD45-BUV395 (clone: HI30; 563792; BD Biosciences); verified reactivity: human (QC testing); application: flow cytometry (routinely tested). Dilution: 1:33.
• Mouse anti-human CD38-BUV737 (clone: HB7; 612824; BD Biosciences); verified reactivity: human (QC testing); application: flow cytometry (routinely tested). Dilution: 1:33.
• Mouse anti-human CD90-APC (clone: 5E10; 559869; BD Biosciences); verified reactivity: human (QC testing), rhesus, cynomolgus, baboon, pig, and dog (tested in development); application: flow cytometry (routinely tested). Dilution: 1:33.
• Mouse anti-human CD135-PE (clone: BV10A4H2; 313306; BioLegend); verified reactivity: human; application: flow cytometric analysis of antibody surface-stained cells. Dilution: 1:33.
• Mouse anti-human CD11c-BV650 (clone: B-ly6; 563404; BD Biosciences); verified reactivity: human (QC testing); application: flow cytometry (routinely tested). Dilution: 1:20.
• Mouse anti-human CD10-BV786 (clone: HI10a; 564960; BD Biosciences); verified reactivity: human (QC testing), rhesus, cynomolgus, and baboon (tested in development); application: flow cytometry (routinely tested). Dilution: 1:20.
• Mouse anti-human CD34-BV421 (clone: 561; 343610; BioLegend); verified reactivity: human; application: flow cytometric analysis of antibody surface-stained cells. Dilution: 1:20.
• Mouse anti-human CD45RA-APCH7 (clone: HI100; 304128; BioLegend); verified reactivity: human; reported reactivity: chimpanzee; application: flow cytometric analysis of antibody surface-stained cells. Dilution: 1:20.
• Mouse anti-human CD71-BV711 (clone: M-A712; 563767; BD Biosciences); verified reactivity: human (QC testing); application: flow cytometry (routinely tested). Dilution: 1:20.
• Mouse anti-human CD19-APCR700 (clone: SJ25C1; 659121; BD Biosciences); verified reactivity: human; application: flow cytometry. Dilution: 1:20.

All the antibodies were purchased from BioLegend and BD Biosciences, and they are well characterized and validated by providers. Titration assays were performed to assess the best antibody concentration. After surface marking, the cells were incubated with PI (BioLegend) to stain dead cells. The absolute cell count was obtained by adding Precision Count beads (BioLegend), fluorescent particles of about 10 μm that can be detected across a broad range of wavelengths (405–635 nm excitation and 400–800 nm emission). A predetermined volume of beads was added to the analyzed samples before any manipulation foreseen by the staining procedures (RBC lysis, filtering, and centrifugation), and the absolute numbers of cells can be determined by comparing the bead count and the cell count. Specifically, according to the manufacturer’s instruction, we calculated the number of cells of interest (expressed as cells/μl) with the following formula: {[(number of events of the cells of interest) × (volume of beads added to the samples)]/[(number of events of count beads) × (volume of samples)]} × concentration of bead count = absolute number of cells of interest (cells/μl).

All stained samples were acquired through BD LSR-Fortessa (BD Biosciences) cytofluorimeter after Rainbow beads (Spherotech) calibration. Raw data were collected through DIVA software version 8.0.2 and analyzed with FlowJo software version 10.5.3 (BD Biosciences). Technically validated results were always included in the analyses, and we did not apply any exclusion criteria for outliers.

### Whole-exome sequencing analysis for identification of CH-associated mutations

Somatic variant calling was performed according to the GATK “Best Practice Workflows” to identify somatic variants in each sample. Identified variants were then confirmed using VarDict (Lai et al., 2016). Raw read quality was assessed using FastQC (version 0.11.9). Raw reads were then trimmed using Trim Galore (version 0.6.6) to remove low-quality bases. To eliminate possible mouse contamination, a disambiguation procedure was performed (Ahdesmäki et al., 2016): reads were aligned to both human and mouse reference genomes, and then each one was assigned to the organism with the best mapping (or marked as ambiguous and discarded). Next, human-specific reads were aligned against the human genome assembly (GRCh38) using BWA (version 0.7.17) (Li and Durbin, 2009). Duplicates were marked using Picard MarkDuplicates (version 2.25.6), and GATK (version 4.2.0.0) BaseRecalibrator and ApplyBQSR were used to recalibrate base quality scores using dbSNP known sites (Smigielski et al., 2000).

Mutect2 in tumor-only mode was used to call variants in each sample with a minimum tumor limit Of detection (LOD) of 2. Variants were then filtered using FilterMutectCalls. Only variants passing all default Mutect2 filters or only failing the “clustered_events” filter were retained for further analyses. Variants were additionally filtered by depth, retaining only variants with a minimum of five variant reads for single nucleotide variants (SNVs) or a minimum of 10 variant reads for indels. Finally, to enrich for true somatic variants, putative germline variants were flagged and removed according to the following criteria: variant alleles with a minor allele frequency >1% in the dbSNP database and with a VAF between 0.4 and 0.6 or >0.9 (Smigielski et al., 2000)

To obtain a high-confidence list of mutations, variants were also independently called using VarDict with a minimum VAF of 0.01, minimum base quality score of 25, and a minimum of two supporting reads (Lai et al., 2016). Variants were filtered by retaining only those with a minimum of five variant reads for SNVs, a minimum of 10 variant reads for indels, a minimum base quality score of 30, a minimum mapping quality score of 40, and a maximum strand bias Fisher P value of 0.0001. To identify a high-confidence set of somatic variants, only variants independently identified by both Mutect2 and VarDict were retained. The high-confidence set of somatic variants in the cohort was then explored using a gene panel containing 97 genes related to CH retrieved from Jakobsen et al. (2024). Somatic variants in CH genes were explored using an OncoPrint reporting the type (SNV, insertion, or deletion) as well as the identity of genes harboring a mutation, with the relative percentage in the cohort under study. OncoPrint were generated using the ComplexHeatmap R package (Gu et al., 2016).

### Limiting dilution xenotransplantation study of uncultured human BM HSPCs across aging

All animal experiments were done in accordance with institutional guidelines approved by the UHN Animal Care Committee. Aged matched female NSG mice (NOD.Cg *Prkdc*scid*Il2rg*tm1Wjl/SzJ; Jackson Laboratory), 10–12 wk of age, were sublethally irradiated with 225 rad 1 day before intrafemoral injection. CB CD34-enriched cells and BM samples were thawed, and CD34 enrichment from BM samples was performed by positive selection with the CD34 Microbead kit (Miltenyi) with MACS magnet technology (Miltenyi) the morning of xenotransplantation. A limiting dilution assay (LDA) to quantitatively measure CD34 repopulating frequency across aging (CB, young BM [21–32 year, *n* = 5], middle-aged BM [52–57 year, *n* = 3], and old BM [78–83 year, *n* = 5] in 314 NSG mice was designed based on a prior study to measure CB functional HSCs frequency in NSG (Laurenti et al., 2015). CD34-enriched samples were injected into the right femur of mice at three cell doses (60,000 cells, 10,000 cells, and 1,000 cells). Flow cytometry analysis was conducted on an aliquot of each sample to quantify the number of CD34^+^ cells injected using the following panel: PE-anti-CD19, APC-anti-CD34, PECy7-anti-CD38, FITC-anti-CD45RA, and propidium iodide. Each sample was distributed across the indicated cell doses for transplant (*n* = 3–11 mice/cell dose) based on available sample material (see Table S1). Human engraftment in the bones of mice was assessed at 4, 12, and 20 wk after transplantation as previously described by flow cytometry analysis on a BD Celesta with the following antibodies: PE-Cy5-anti-CD45, V500-anti-CD45, PE-anti-GlyA, PE-anti-CD19, BV786-anti-CD33, APC-anti-CD34, FITC-anti-CD71, APC-Cy7-anti-CD41, and BV605-anti-CD56 and a viability dye (Laurenti et al., 2015; Xie et al., 2019) (sytox blue, Thermo Fisher Scientific). Mice were considered engrafted if CD45^+^ cells were >0.05%. CD34 repopulating frequency was estimated using ELDA software (Hu and Smyth, 2009) (http://bioinf.wehi.edu.au/software/elda/).

### *In vitro* multi-lineage differentiation assay

For differentiation assays, 500 Lin^−^CD34^+^ cells (bulk), 500 HSCs (Lin^−^CD34^+^CD38^−^CD90^+^CD45RA^−^ cells), or single HSCs (Lin^−^CD34^+^CD38^−^CD90^+^CD45RA^−^ cells, also stained for CD49f to perform index sorting analyses) were sorted from young (*n* = 3) or old (*n* = 3) subjects and seeded on non-tissue culture–treated flat-bottom plates (Thermo Fisher Scientific) pre-coated with StemSpan Differentiation Coating Material (Stem Cell Technologies), according to the manufacturer’s specifications. Cells were cultured in SFEM II medium (Stem Cell Technologies) supplemented with hSCF (100 ng/ml), hFLT3 (10 ng/ml), hIL-7 (100 ng/ml), hIL-2 (10 ng/ml) (Novartis), hTPO (75 ng/ml), hIL-6 (40 ng/ml), hIL-3 (10 ng/ml), hIL-11 (50 ng/ml), hEPO (0.1 U/ml) (PeproTech), hIL-4 (10 ng/ml) (Miltenyi Biotec), and hLDL (4 µg/ml) (Stem Cell technologies). Medium change was performed every 3–4 days. After 3 wk of culture, cells were harvested and labeled with the following anti-human–conjugated antibodies: anti-CD235a, anti-CD1a, anti-CD5, anti-CD19, anti-CD42b, CD33, anti- CD7, anti-CD71, and anti-CD11c (BD Bioscience) and anti-CD41, anti-CD10, anti-CD15, anti-CD3, anti-CD56, and anti-CD34 (BioLegend). The stained samples were acquired through BD FACS Symphony A5 (BD Biosciences) cytofluorimeter after Rainbow bead (Spherotech) calibration. Raw FACS data were collected through DIVA software (BD Biosciences) and subsequently analyzed with FlowJo software version 10.5.3, and the graphical output was generated through Prism version 10.0.0 (GraphPad). The threshold for positive wells was determined based on negative controls. To annotate the potential of LT-HSC, each single-cell output was scored as uni-, bi-, and multi-lineage based on the most abundant differentiation output toward lymphoid, myeloid, erythroid, and megakaryocyte lineages. Uni-lineage clones were defined when >75% of produced cells belonged to only one lineage, while bi-lineage clones were characterized by two major differentiation outputs (each >20%) accounting for >70% of total cell output. Cells with detectable output toward all the four lineages were defined as multi-lineage, while cells with heterogeneous output, not falling in the other three categories, were defined as mixed clones.

### Generation of scRNAseq atlas and analysis

We combined publicly available scRNAseq dataset profiling BM-derived HSPCs collected from young (16–30 years) or elderly (>60 years) human healthy donors, namely: Ainciburu et al. (GSE180298), Li et al. (GSE189161), Mende et al. (GSE190067), Quaranta et al. (GSE253485), and Setty et al. (European Nucleotide Archive [ENA] ERP120467). Such combined dataset profiles the transcriptomes of 134,184 cells across 13,748 genes, upon the removal of donors with <100 sequenced cells and sequenced cells with <500 or >5,000 detected genes. We set up a standardized analysis using Seurat R package version 4.1.1 (Butler et al., 2018; Stuart et al., 2019) and harmony R package version 0.1. The expression level of each gene was then scaled (ScaleData) by its overall library size (nCount_RNA), and the scaled gene expression matrix was then fed as an input to compute the principal components (RunPCA) restricting the analysis to the top 1,000 most variable genes (NormalizeData and FindVariableFeatures). The first 67 PCs were then used to perform data integration with Harmony (RunHarmony) accounting for both dataset and donor batch effects. The resulting harmonized PCs were then used to compute the harmonized UMAP embeddings (RunUMAP) and to define a neighboring cell-graph (FindNeighbors). Such graph was then used to perform cell clustering (FindClusters) at different resolutions (0.1 to 1 by 0.1) through the Louvain algorithm. Module scores (AddModuleScore) were computed based on a set of cell type–specific markers derived from previous publications. Such modules combined with the results of between-clusters differential gene expression (DGE) analyses were used to associate each inferred cell cluster (res = 0.5) to a cell type label. Finally, we performed subclustering (FindSubCluster, res = 0.2) on the most primitive HSPCs set (namely, HSCs) and identified subsets of cells showing LT-HSCs, MPP, or lineage-primed profiles. To infer the cell cycle, a continuous measure was derived by the CellCycleScoring R function based on the “regev_lab_cell_cycle_human.rds” cell cycle marker genes. This classification was further refined computing cell-wise module scores based on sets of genes that positively or negatively regulate dormancy, allowing classification of G0 cells within G1 cells showing a highly dormient transcriptomic profile. DGE analysis between two groups of cells was performed (FindMarkers) through a Wilcoxon Mann–Whitney test. The nominal P values were adjusted to account for the multiple hypotheses testing issue through the Benjamini–Hochberg procedure, and the difference in expression levels of each gene or gene set was deemed as significant whenever the adjusted P value was smaller or equal to 0.05.

### ATAC-seq library preparations and analyses

#### From human HSPCs subpopulations

Library preparation for ATAC-seq was performed on 1,000–2,000 cells with the NextEra DNA Sample Preparation kit (Illumina), according to previously reported protocol (Buenrostro et al., 2013). Four ATAC-seq libraries were sequenced per lane in the HiSeq 2500 System (Illumina) to generate paired-end 50-bp reads. Raw sequencing reads were aligned to the hg38 genome build using BWA (Li and Durbin, 2009). All duplicate reads and reads mapped to mitochondria, chrY, an ENCODE blacklisted region, or an unspecified contig were removed (ENCODE Project Consortium, 2012). The set of samples (*n* = 31 subfractions, *n* = 3 young donors [21–28 years], *n* = 4 mid/old donors [52–79 years]) that passed quality control and were used for downstream analysis is in Table S3. The catalog of all peaks called in any population was produced by merging all called peaks that overlapped by at least one base pair using bedtools and dividing into 500-bp windows. The MACS bdgcmp function (Zhang et al., 2008) was used to compute the fold enrichment over background for all populations, and the bedtools map function was used to identify the maximum fold enrichment observed at each window covering a peak in the catalog in each population. Maximum fold enrichment at each site in the catalog was quantile normalized between samples; we define this as the ATAC-seq signal for further analyses. The NMF package (Gaujoux and Seoighe, 2010) was used to perform NMF on the quantile normalized fold enrichment at each peak in the catalog (using the Brunet method with default parameters) for between 3 and 10 signatures. 6 signatures were selected as optimal. For each signature, sites were ranked according to the strength of each signature, and the top 5% of sites were identified as being the sites for each signature. Homer (Heinz et al., 2010a) was used to identify uniquely enriched motifs within the top 5% of sites for each signature, using default parameters. Contiguous genomic windows were merged, and the catalog of all called peaks was used as a background. Sites enriched in the top 5% of sites of both signatures were removed prior to computing enrichment.

#### From xenotransplantation model of proliferative stress

A total of 1 × 10^5^ cells from purified human CD34^+^ from murine BM samples were lysed with digitonin (cat. #G944A; Promega) and tagmented with an engineered Tn5 transposase (cat. #15027865; Illumina) at 37°C for 30 min, following a protocol optimized for blood cells. Tagmented DNA was purified and amplified with 10 cycles of PCR. Before the sequencing, fragments with a 1–5 kbp size range were removed by magnetic separation with AMPure XP beads (cat. #A63881; Beckman Coulter). For the downstream analyses, reads were trimmed with Trim Galore, aligned with BWA-MEM (release 0.7.17-r1188), to the human genome (GRCh38 primary assembly). *Mus musculus* (GRCm39) and *Bos taurus* (GCF_002263795.3) genomes were used for sequential disambiguation (Ahdesmäki et al., 2016) to remove potential contaminant reads from FBS and host DNA of the *in vivo* experiment. Bam files were filtered for mitochondrial, Y chromosome and PCR duplicated reads with Picard (2.25.0-0). Blacklisted regions were filtered using as reference ENCODE blacklist (hg38 version 2). Soft-clipped reads were filtered out with gatk when the ratio of clipped over the total bases exceeded 0.25. Finally, only properly paired reads, with non-secondary alignment and mapping quality of at least 15 and length >45 bp were retained. To quantify open chromatin regions, bedtools multibamcov (with parameters -q 10) was used against the Rank6 signatures regions unique of old and young conditions, generated from the previous experiment. Differential accessibility was computed with DESeq2, filtering regions with less than 10 counts, and testing for the contrast high vs low dose (FDR 0.05). The analysis of known motif enriched in DARs was conducted with Homer software (Heinz et al., 2010b) (parameters -size 200 -mask).

### Total RNAseq library preparation and analysis

Whole transcriptomic analysis was performed on a pool of BM CD34^+^ cells derived from three young and old donors. All conditions were performed in triplicate. Total RNA was isolated after an overnight in liquid culture using the miRNeasy Micro Kit (Qiagen), and the DNase treatment was performed using RNase-free DNase Set (Qiagen), according to the manufacturer’s instructions. Total RNA was used for library preparation with the NEBNext Ultra-low input II Directional RNA Library Prep Kit (Illumina) and sequenced on an Illumina HiSeq with a 2 × 150 bp sequencing configuration (Illumina).

### RNAseq analysis

High-quality reads, obtained after read quality inspection and adapter trimming with Trim Galore (https://www.bioinformatics.babraham.ac.uk/projects/trim_galore/), were aligned to the human reference genome (GENCODE GRCh38 primary assembly) with STAR release 2.7.6a. Gene expression quantification was obtained with featureCounts version 2.0.1, using the GENCODE annotation (version 35 primary assembly). Raw counts were normalized by library size, and the DE analysis was performed with the DESeq2 package, testing for the contrast old vs. young. Finally, Benjamini–Hochberg adjusted P values were used to retain significant DEGs (FDR < 0.05). For the GSEA (Subramanian et al., 2005), genes were ranked by log-fold change and analyzed with the gsePathway function from clusterProfiler, considering the Reactome Database, retaining gene categories with FDR < 0.05. All figures were plotted with ggplot2 in R.

### CFU assay

CFU assay was performed at the indicated time points, plating 800 cells in methylcellulose-based medium (MethoCult H4434, StemCell Technologies) supplemented with penicillin and streptomycin. 2 wk after plating, colonies were counted in blind and identified as myeloid and erythroid colonies according to morphological criteria.

### *In vitro* growth curve of young and old CD34^+^ cells

Human CD34^+^ cells were seeded at the concentration of 5 × 10^5^ CD34^+^ cells/ml in serum-free StemSpan medium (StemCell Technologies) supplemented with penicillin, streptomycin, glutamine, and human early-acting cytokines (SCF 300 ng/ml, Flt3-L 300 ng/ml, TPO 100 ng/ml, and IL-3 60 ng/ml; all purchased from Peprotech). All cultures were kept at 37°C in a 5% CO_2_ water jacket incubator (Thermo Fisher Scientific), and cell count was assessed every 24 h.

### Ki67 staining and flow cytometry analysis

Cells were washed and fixed using BD Cytofix buffer (Cat. #554655), washed and permeabilized with BD Perm 2 (cat. #347692), and washed and stained with FITC-conjugated Ki67 antibody (556026: BD Biosciences). The cells were then analyzed on a BD LSR-Fortessa cytometer (BD Biosciences).

### CellTrace proliferation assay

Bulk HSPCs or FACS-sorted HSPCs subsets from young and aged healthy donors were washed and incubated with CellTrace Violet (C34557; Thermo Fisher Scientific) 5 mM for 20 min at 37°C, protected from light. Cell sorting was performed using FACS Aria Fusion (BD Biosciences). After incubation, cells were washed and resuspended in a fresh pre-warmed complete culture medium and cultured in serum-free StemSpan medium (StemCell Technologies) supplemented with penicillin, streptomycin, glutamine, and hSCF 300 ng/ml, hFlt3-L 300 ng/ml, hTPO 100 ng/ml, and hIL-3 60 ng/ml (all purchased from Peprotech). Cells were collected every 24 h to measure the cell divisions through flow cytometry. Cells were acquired using a BD LSR-Fortessa Cytometer (BD Biosciences).

### Gene expression analysis

For gene expression analyses, total RNA was extracted using either the miRNeasy Micro Kit (Qiagen) or the RNeasy Plus Micro Kit (Qiagen), according to the manufacturer’s instructions, and DNase treatment was performed using the RNase-free DNase Set (Qiagen). cDNA was synthetized with the iScript cDNA Synthesis Kit (Bio-Rad). For selected analyses, cDNA was then pre-amplified using TaqMan PreAmp Master Mix (Thermo Fisher Scientific) and used for q-PCR in a Viia7 Real-time PCR thermal cycler using Fast SYBR Green Master Mix (Thermo Fisher Scientific), after standard curve method optimization to reach the 100% primer efficiency for each couple of primers listed in Table S7. The relative expression of each target gene was first normalized to *GUSB* or β*2M* housekeeping gene expression and then represented as 2^−ΔCt^ relative to the indicated control conditions.

### Immunofluorescence analysis

Multi-test slides (10 well; MP Biomedicals) were treated for 20 min with Poly-L-lysine solution (Sigma-Aldrich) at 1 mg/ml concentration. After two washes with DPBS solution, ∼0.5/1 × 10^5^ cells were seeded on covers for 20′ and fixed with 4% paraformaldehyde (Santa Cruz Biotechnology) for the other 20′. Cells were then permeabilized with 0.2% Triton X100. After blocking with 0.5% BSA and 0.2% fish gelatin in DPBS, cells were probed with the indicated primary antibodies. After primary antibody incubation (Anti-phospho RPA [Ser33] Antibody; Merck), cells were washed three times with DPBS and incubated with Alexa 488-, 568-, and/or 647-labeled secondary antibodies (Invitrogen). Nuclear DNA was stained with DAPI (Sigma-Aldrich), and covers were mounted with Aqua-Poly/Mount solution (Polysciences, Inc.) on glass slides (Bio-Optica). Fluorescent images were acquired using Leica SP2 and Leica SP5 confocal microscopes. Where indicated, quantification of DNA damage response nuclear foci in immunofluorescence images was conducted using ImageJ64 (version 1.47).

### Transplantation experiments of activated human HSPCs across aging and for the *in vivo* model of proliferation stress

Mouse studies were conducted according to protocols approved by the San Raffaele Scientific Institute and the Italian Ministry of Health (#872; IACUC). NOD.Cg-Prkdc^scid^ IL2rγ^tm1Wjl^/SzJ (NSG) mice were purchased from The Jackson Laboratory (cat. #005557; Jackson Laboratories). All animals were maintained in the SPF animal facility at IRCCS Ospedale San Raffaele. The handling of the animals was performed by trained personnel, with the aim of minimizing the degree of stress and suffering and the period of constraint.

For transplantation, 2.5 × 10^5^ BM-CD34^+^ cells isolated from young and old healthy donors or different doses of CB-CD34^+^ (50 × 10^3^; 10 × 10^3^; 3 × 10^3^) were injected intravenously into 8-wk-old female sublethally irradiated NSG mice (150–180 cGy). Mice were monitored three times a week by weight assessment and general appearance, taking into consideration motility, fur conditions, kyphosis, and other signs of disease. Human CD45^+^ cell engraftment and blood lineage reconstitution were monitored by serial collection of blood from the mouse tail, and at the time of sacrifice, BM, spleen, and distal organs were harvested. Human cell engraftment in murine organs was assessed by applying the WBD protocol (see WBD staining section). Before the WBD procedure, cells were incubated with a mouse FcR blocking reagent (BD Biosciences, dilution 1:100). Mice with human cell engraftment below 0.05% (in primary transplants) and 0.005% (in secondary transplants) in the murine BM were considered not engrafted. This threshold was based on the analyses of mice not transplanted with human cells and it was in line with the commonly used threshold for human xenotransplants (Doulatov et al., 2012).

### BrdU and EdU *in vivo* administration and staining procedure

For cell proliferation analysis, EdU and BrdU double labeling was used as previously described (Akinduro et al., 2018). Both nucleotide analogs are incorporated during DNA synthesis and, when combined, allow the analysis of cell cycle kinetics. Pulse-chase timings were previously optimized (Akinduro et al., 2018) to achieve consistent *in vivo* incorporation of EdU and BrdU and later detection in the cell population of interest. EdU (1 mg/mouse, Component A, Click-iT EdU Alexa Fluor 647; C10635; Thermo Fisher Scientific) and BrdU (2 mg/mouse; B5002-100MG; Sigma-Aldrich) were subsequently administrated via tail vein injections (2 h apart); 30 min after BrDU injection, mice were culled, and BM cells were isolated to perform human cell staining. The staining procedure was performed as follows.

• Surface staining of BM cells labeled with the following fluorescent antibodies:
• Mouse anti-human CD45-BUV395 (clone: HI30; 563792; BD Biosciences); verified reactivity: human (QC testing); application: flow cytometry (routinely tested). Dilution: 1:33.
• Mouse anti-human CD3-BV605 (clone: OKT3; 317322; BioLegend); verified reactivity: human; application: flow cytometric analysis of antibody surface-stained cells. Dilution: 1:50.
• Mouse anti-human CD56-PC5 (clone: 5.1H11; 362516; BioLegend); verified reactivity: human; application: flow cytometric analysis of antibody surface-stained cells. Dilution: 1:50.
• Mouse anti-human CD14-PE (clone: M5E2; 301806; BioLegend); verified reactivity: human, cynomolgus, and rhesus; application: flow cytometric analysis of antibody surface-stained cells. Dilution: 1:50.
• Mouse anti-human CD34-BV421 (clone: 561; 343610; BioLegend); verified reactivity: human; application: flow cytometric analysis of antibody surface-stained cells. Dilution: 1:20.
• Mouse anti-human CD15-APCfire750 (clone: W6D3; 323041; BioLegend); verified reactivity: human; application: flow cytometric analysis of antibody surface-stained cells. 1:50.
• Mouse anti-human CD19-APCR700 (clone: SJ25C1; 659121; BD Biosciences); verified reactivity: human; application: flow cytometry. Dilution: 1:20.
• Mouse anti-human CD38-BV510 (clone: HB-7; 356612; BioLegend); verified reactivity: human (QC testing); application: flow cytometry (routinely tested). Dilution: 1:40.
• Mouse anti-human CD11c-BV650 (clone: B-ly6; 563404; BD Biosciences); verified reactivity: human (QC testing); application: flow cytometry (routinely tested). Dilution: 1:20.
• Fixation and permeabilization followed by EdU staining (Click-iT EdU Alexa Fluor 647, C10635; Thermo Fisher Scientific) according to the manufacturer’s instructions.
• Permeabilization using Cytoperm Buffer PLUS (BD Biosciences) 10 min on ice followed by permeabilization through CytoFix/CytoPerm kit solution (BD Biosciences) 5 min at room temperature (RT).
• DNAse treatment (Qiagen) for 1 h at 37°C.
• Staining with anti-BrDU-Alexa488 antibody, clone: 3D4, BioLegend; 364105; application: intracellular flow cytometry (routinely tested). Dilution: 1:50.

Samples were acquired at BD LSR-Fortessa (BD Biosciences) at a 400 event/sec rate.

### Neutral and alkaline comet assays

Purified HSPCs from CD34^+^ cells isolated from mice transplanted with distinct doses of CD34^+^ cells at 20 wk were suspended at 1 × 10^5^ cells/ml in ice-cold 1X DPBS and mixed with molten Comet LMAgarose (Trevigen) at a ratio of 1:10 (vol/vol) and immediately pipetted onto CometSlides (Trevigen) and placed at +4°C. Once solidified, the slides were immersed in pre-chilled Lysis Solution (MD) overnight at +4°C. For the neutral comet assay, after lysis, slides were immersed in neutral electrophoresis buffer, pH 8 (50 mM Tris and 150 mM sodium acetate) for 1 h at +4°C and then electrophoresed in neutral electrophoresis buffer, pH 8 (100 mM Tris and 300 mM sodium acetate) at 300 mA for 40 min. Slides were gently immersed in DNA precipitation solution (375 mM ammonium acetate in 95% ethanol) for 30 min at RT and fixed in 70% ethanol for 30 min. For the alkaline comet assay, after lysis, slides were immersed in freshly prepared alkaline unwinding solution, pH > 13 (300 mM NaOH and 1 mM EDTA) for 1 h at +4°C and then electrophoresed in alkaline electrophoresis solution, pH > 13 (300 mM NaOH and 1 mM EDTA) at 300 mA for 20 min. Slides were washed twice in ddH_2_O and fixed in 70% ethanol for 5 min. Comets were stained with SYBR Safe (Invitrogen) for 30 min at RT. All steps were conducted in the dark to prevent additional DNA damage. Comets were analyzed using a Nikon Eclipse E600 microscope and a Nikon-DS-RI2 camera. At least 80 nuclei for each individual donor were analyzed with CASP software to determine “Olive Tail Moments” of individual nuclei.

### Statistical analyses

Statistical tests and P values were specified in each figure legend or within figure graphs. To assess the correlation between two variables, the Spearman index was calculated, and a regression line (with 95% confidence intervals) was estimated after assessing the normality of residuals as appropriate. Nonparametric tests were used for comparing two or more groups when continuous variables were considered. The analyses were performed using Prism version 9.1.0 software (GraphPad). For the longitudinal analyses of the growth curve of cultured cells, a linear mixed-effects (LME) model has been applied. To better capture growth dynamics, the group indicator (young vs*.* old) and the time variable (a categorical variable taking five values, from 0 to 96 h) were entered in the model as main effects as well as in interaction. To account for donor-specific heterogeneity, a random effect was specified on donors’ IDs, thus leading to estimated random intercept models. This modeling approach properly accounts for the dependency among observations arising from measuring the same unit more than once and over time. Logarithmic transformation of the outcome variable was considered to satisfy underlying model assumptions. Analyses were performed using R statistical software (version 4.0.4). The significance level was set at 0.05. LME were estimated using the nlme package, and post hoc comparisons to compare groups at a fixed time point have been performed using the emmeans package.

### Online supplemental material

Figs. S1, S2, and S3 support the data regarding the steady-state phenotypic composition of human BM and PB samples and human HSPCs repopulating potential in xenotransplantation assay across age. Fig. S1 shows the gating strategy used for the identification of HSPCs subpopulations and the correlation between the absolute count and the age of each cell subtype in the BM of the 73 subjects analyzed. Fig. S2 shows the correlation between the absolute count and the age of each cell subtype in the PB of the 73 subjects analyzed and the CH-associated mutations analysis in aged BM samples via whole-exome sequencing. Fig. S3 shows the frequency of distinct HSPCs subpopulations in healthy donors from different ages in the Italian cohort and in the North American (Canada) cohort. Moreover, the figure shows *in vivo* reconstitution of B and myeloid cells in the murine BM upon primary and secondary transplantations of CB and BM human samples at different time points. Fig. S4 supports the findings regarding the human-aged HSPCs proliferation kinetics upon *ex vivo* activation and their differentiation potential after in *vivo* transplantation. The figure shows DEGs between old and young HSPCs after pre-activation and their associated categories. Moreover, the figure shows the *in vitro* cell division analysis of young and old CD34^+^ cells over time and the *in vivo* reconstitution capacities in the BM and PB of mice transplanted with pre-activated young and aged HSPCs. Fig. S5 supports the *in vivo* model of proliferation stress by low cell dose transplantation. The figure shows the composition of human HSPCs compartment in the BM of mice transplanted with different cell doses and the correlation between the percentage of proliferating HSPCs and different level of engraftment in the murine BM. Moreover, the figure shows genomic and DNA damage analysis of CD34^+^ cells isolated from mice transplanted with different cell doses. Table S1 provides sample information for immunophenotypic analysis for the Italian cohort. Table S2 provides CD34-enriched sample characteristics and limiting dilution xenotransplantation data estimating CD34^+^ repopulating frequency across human ages. Table S3 provides annotated clusters per dataset. Table S4 provides sample information for bulk ATAC-seq NMF analysis. Table S5 provides ATAC-seq enrichment data for all motifs identified. Table S6 provides DEGs and GSE-Reactome data. Table S7 provides RT-qPCR primers list.

## Supplementary Material

Table S1 shows sample information for immunophenotypic analysis for the Italian cohort.

Table S2 shows xenotransplantation sample metadata.

Table S3 shows annotated clusters per dataset.

Table S4 shows sample information for bulk ATAC-seq NMF analysis.

Table S5 provides ATAC-seq enrichment data for all motifs identified.

Table S6 shows DGE analysis results of old vs young at steady state (DESeq2).

Table S7 shows RT-qPCR primers.

## Data Availability

All the data generated in this study have been deposited in the San Raffaele Open Research Data Repository under accession code https://doi.org/10.17632/72ty5v9djn.1. The RNA- and ATAC-sequencing data generated and discussed in this study have been deposited in NCBI’s Gene Expression Omnibus (Edgar et al., 2002) and are accessible through GEO SuperSeries GSE311225, which contains the following data: GSE243327 (RNAseq) and GSE311221 (ATAC-seq). The code used to process and to generate the images of RNAseq data in this manuscript is available at: http://www.bioinfotiget.it/gitlab/custom/LetteraScala_Ageing. WES data generated and discussed in this study are available at the ENA under the following accession code: PRJEB93902.
