# Supplementary material for: Molecular and phenotypic blueprint of human hematopoiesis links proliferation stress to stem cell aging
Source: J Exp Med. 2025 Dec 30;223(2):e20251805. doi: 10.1084/jem.20251805 (PMC13248933; doi:10.1084/jem.20251805)
Supplement: Table S1 — shows sample information for immunophenotypic analysis for the Italian cohort. [file jem_20251805_tables1.docx]

**Table S1. Sample information for immunophenotypic analysis for the Italian cohort**

N.A. = information not available

| **Pediatric Group** |  |  |
| --- | --- | --- |
| **ID sample** | **Tissue Type** | **Age (y)** |
| HD PED 1 | BM | 10 |
| HD PED 2 | BM | 15 |
| HD PED 3 | BM | 17 |
| HD PED 4 | BM | 6 |
| HD PED 5 | BM | 11 |
| HD PED 6 | BM | 3 |
| HD PED 7 | BM | 12 |
| HD PED 8 | BM | 5 |
| HD PED 9 | BM | 9 |
|  |  |  |
| **Young-Adult Group** |  |  |
| **ID sample** | **Tissue Type** | **Age (y)** |
| HD AD 1 | BM | 28 |
| HD AD 2 | BM | 26 |
| HD AD 3 | BM | 18 |
| HD AD 4 | BM | N.A. |
| HD AD 5 | BM | 19 |
| HD AD 6 | BM | 24 |
| HD AD 7 | BM | 30 |
| HD AD 8 | BM | 29 |
| HD AD 9 | BM | 35 |
| HD AD 10 | BM | 39 |
| HD AD 11 | BM | 29 |
| HD AD 12 | BM | 36 |
| HD AD 13 | BM | 22 |
|  |  |  |
|  |  |  |
| **Middle Aged** |  |  |
| **ID sample** | **Tissue Type** | **Age (y)** |
| HD MA 1 | BM | 41 |
| HD MA 2 | BM | 56 |
| HD MA 3 | BM | 63 |
| HD MA 4 | BM | 49 |
| HD MA 5 | BM | 62 |
| HD MA 6 | BM | 57 |
| HD MA 7 | BM | 61 |
| HD MA 8 | BM | 64 |
| HD MA 9 | BM | 50 |
| HD MA 10 | BM | 48 |
| HD MA 11 | BM | 47 |
| HD MA 12 | BM | 42 |
| HD MA 13 | BM | 43 |
| HD MA 14 | BM | 42 |
| HD MA 15 | BM | 55 |
| HD MA 16 | BM | 56 |
| HD MA 17 | BM | 58 |
| HD MA 18 | BM | 55 |
| HD MA 19 | BM | 62 |
| HD MA 20 | BM | 53 |
|  |  |  |
| **OLD** |  |  |
| **ID sample** | **Tissue Type** | **Age (y)** |
| HD OLD 1 | BM | 75 |
| HD OLD 2 | BM | 73 |
| HD OLD 3 | BM | 84 |
| HD OLD 4 | BM | 78 |
| HD OLD 5 | BM | 74 |
| HD OLD 6 | BM | 87 |
| HD OLD 7 | BM | 80 |
| HD OLD 8 | BM | 72 |
| HD OLD 9 | BM | 76 |
| HD OLD 10 | BM | 82 |
| HD OLD 11 | BM | 73 |
| HD OLD 12 | BM | 76 |
| HD OLD 13 | BM | 69 |
| HD OLD 14 | BM | 77 |
| HD OLD 15 | BM | 80 |
| HD OLD 16 | BM | 68 |
| HD OLD 17 | BM | 74 |
| HD OLD 18 | BM | 65 |
| HD OLD 19 | BM | 74 |
| HD OLD 20 | BM | 69 |
| HD OLD 21 | BM | 69 |
| HD OLD 22 | BM | 67 |
| HD OLD 23 | BM | 68 |
| HD OLD 24 | BM | 65 |
| HD OLD 25 | BM | 78 |
| HD OLD 26 | BM | 88 |
| HD OLD 27 | BM | 70 |
| HD OLD 28 | BM | 72 |
| HD OLD 29 | BM | 67 |
| HD OLD 30 | BM | 83 |
| HD OLD 31 | BM | 69 |

| **Pediatric Group** |  |  |
| --- | --- | --- |
| **ID sample** | **Tissue Type** | **Age (y)** |
| HD PED 1 | PB | <1 |
| HD PED 2 | PB | <1 |
| HD PED 3 | PB | <1 |
| HD PED 4 | PB | <1 |
| HD PED 5 | PB | 1 |
| HD PED 6 | PB | 1 |
| HD PED 7 | PB | 7 |
| HD PED 8 | PB | 7 |
| HD PED 9 | PB | 7 |
| HD PED 10 | PB | 10 |
| HD PED 11 | PB | 12 |
| HD PED 12 | PB | 18 |

| **Young-Adult Group** |  |  |
| --- | --- | --- |
| **ID sample** | **Tissue Type** | **Age (y)** |
| HD AD 1 | PB | 30 |
| HD AD 2 | PB | 37 |
| HD AD 3 | PB | 30 |
| HD AD 4 | PB | 32 |
| HD AD 5 | PB | 26 |
| HD AD 6 | PB | N.A |
| HD AD 7 | PB | N.A |
| HD AD 8 | PB | N.A |
| HD AD 9 | PB | 31 |
| HD AD 10 | PB | 28 |
| HD AD 11 | PB | 28 |
| HD AD 12 | PB | 28 |
| HD AD 13 | PB | 37 |
| HD AD 14 | PB | 25 |
| HD AD 15 | PB | 28 |
| HD AD 16 | PB | 26 |
| HD AD 17 | PB | 32 |

| **Middle Aged** |  |  |
| --- | --- | --- |
| **ID sample** | **Tissue Type** | **Age (y)** |
| HD MA 1 | PB | 56 |
| HD MA 2 | PB | 63 |
| HD MA 3 | PB | 60 |
| HD MA 4 | PB | 62 |
| HD MA 5 | PB | 57 |
| HD MA 6 | PB | 61 |
| HD MA 7 | PB | 64 |
| HD MA 8 | PB | 50 |

| **OLD** |  |  |
| --- | --- | --- |
| **ID sample** | **Tissue Type** | **Age (y)** |
| HD OLD 1 | PB | 74 |
| HD OLD 2 | PB | 87 |
| HD OLD 3 | PB | 80 |
| HD OLD 4 | PB | 72 |
| HD OLD 5 | PB | 76 |
| HD OLD 6 | PB | 86 |
| HD OLD 7 | PB | 89 |
| HD OLD 8 | PB | 76 |
| HD OLD 9 | PB | 89 |
| HD OLD 10 | PB | 77 |
| HD OLD 11 | PB | 82 |
| HD OLD 12 | PB | 73 |
| HD OLD 13 | PB | 76 |
| HD OLD 14 | PB | 69 |
| HD OLD 15 | PB | 77 |
| HD OLD 16 | PB | 80 |
| HD OLD 17 | PB | 68 |
| HD OLD 18 | PB | 74 |
| HD OLD 19 | PB | 65 |
