# Supplementary material for: Molecular and phenotypic blueprint of human hematopoiesis links proliferation stress to stem cell aging
Source: J Exp Med. 2025 Dec 30;223(2):e20251805. doi: 10.1084/jem.20251805 (PMC13248933; doi:10.1084/jem.20251805)
Supplement: Table S2 — shows xenotransplantation sample metadata. [file jem_20251805_tables2.docx]

**Table S2. Xenotransplantation sample metadata**

Sample information and immunophenotypic information on the CD34-enriched cells transplanted.

Each transplantation experiment coupled CB to indicated BM age groups (21-32y; 52-57; 77-83y)

| **Expt #** | **Tissue type** | **Gender** | **Age** | **Tissue Source** |  |
| --- | --- | --- | --- | --- | --- |
|  |  |  |  |  |  |
| EXPT1 | CB |  |  | Toronto |  |
|  | BM | M | 32 | Toronto |  |
|  | BM | F | 52 | Mexico |  |
|  | BM | F | 78 | Mexico |  |
| EXPT2 | CB |  |  | Toronto |  |
|  | BM | F | 21 | Lonza |  |
|  | BM | M | 57 | Mexico |  |
|  | BM | F | 79 | Mexico |  |
| EXPT3 | CB |  |  | Toronto |  |
|  | BM | M | Samp | Lonza |  |
|  | BM | F | 55 | Mexico |  |
|  | BM | F | 83 | Mexico |  |
| EXPT4 | CB |  |  | Toronto |  |
|  | BM | F | 32 | Toronto |  |
|  | BM | F | 30 | Toronto |  |
|  | BM | F | 78 | Mexico |  |
|  | BM | F | 77 | Mexico |  |

| **Expt #** | **% of live** | | | | |
| --- | --- | --- | --- | --- | --- |
|  | **CD34+** | **CD34+CD19+** | **CD34+CD19-** | **CD34+CD19-CD38-CD45RA-** | **CD34+CD19-CD45RA+** |
| EXPT1 | 55.4 | 1.11 | 53.5 | 4.67 | 17.9 |
|  | 57.1 | 7.14 | 49.1 | 7.14 | 21.4 |
|  | 84.3 | 8.46 | 74.7 | 4.04 | 51.2 |
|  | 72.4 | 16.9 | 54.7 | 6.97 | 20.6 |
| EXPT2 | 55 | 1.2 | 53.6 | 10.4 | 10.5 |
|  | 81.1 | 32.6 | 47.6 | 1.44 | 19.5 |
|  | 75.9 | 8.33 | 66.8 | 27.6 | 6.29 |
|  | 86.4 | 2.39 | 83.5 | 17.3 | 25.1 |
| EXPT3 | 89.4 | 0.477 | 88.7 | 14.7 | 20.3 |
|  | 35.2 | 3.99 | 30.9 | 3.55 | 11.6 |
|  | 90.5 | 7.36 | 80.7 | 45.2 | 5.83 |
|  | 75.2 | 4.07 | 69.9 | 14.1 | 10.2 |
| EXPT4 | 84.3 | 1.51 | 82.7 | 64.8 | 11.7 |
|  | 63 | 2.63 | 59.8 | 37.4 | 8.08 |
|  | 53.3 | 2.4 | 49.1 | 12.6 | 22.2 |
|  | 80.1 | 35 | 46 | 13.9 | 15.6 |
|  | 69.3 | 4.12 | 64.7 | 23.1 | 16.9 |

| **Expt #** | **% of CD34+** | |  |  |  |
| --- | --- | --- | --- | --- | --- |
|  | **CD34+CD19-CD38-CD45RA-** | **CD34+CD19-CD45RA+** | **# CD34+/60K** | **# CD34+/10K** | **# CD34+/1K** |
| EXPT1 | 8.44 | 32.3 | 33240 | 5540 | 554 |
|  | 12.5 | 37.5 | 34260 | 5710 | 571 |
|  | 4.79 | 60.7 | 50580 | 8430 | 843 |
|  | 9.62 | 28.5 | 43440 | 7240 | 724 |
| EXPT2 | 19 | 19.1 | 33000 | 5500 | 550 |
|  | 1.77 | 24 | 48660 | 8110 | 811 |
|  | 36.4 | 8.29 | 45540 | 7590 | 759 |
|  | 20 | 29 | 51840 | 8640 | 864 |
| EXPT3 | 16.5 | 22.7 | 53640 | 8940 | 894 |
|  | 10.1 | 33.1 | 21120 | 3520 | 352 |
|  | 50 | 6.44 | 54300 | 9050 | 905 |
|  | 18.8 | 13.5 | 45120 | 7520 | 752 |
| EXPT4 | 76.9 | 13.8 | 50580 | 8430 | 843 |
|  | 59.3 | 12.8 | 37800 | 6300 | 630 |
|  | 23.6 | 41.6 | 31980 | 5330 | 533 |
|  | 17.3 | 19.5 | 48060 | 8010 | 801 |
|  | 33.3 | 24.4 | 41580 | 6930 | 693 |

**Limiting dilution xenotransplantation assays to quantitatively measure 1/CD34 repopulating frequency across the human lifetime**

CD34+ enriched samples were transplanted into NSG mice at indicated cell doses into indicated numbers of mice and analyzed at 4, 12 or 20 wks. Engraftment response is defined at >0.05% CD45+.

| **EXPT1** | | | | | |
| --- | --- | --- | --- | --- | --- |
| **Sample** | **#CD34+ cells transplanted** | **# mice tested** | **#responders** | **Cell dose** | **Time point** |
| CB | 33240 | 3 | 3 | 60K | 4 weeks |
|  | 5540 | 3 | 3 | 10K |  |
|  | 554 | 3 | 3 | 1K |  |
| 52y F BM | 50580 | 4 | 4 | 60K |  |
|  | 8430 | 3 | 3 | 10K |  |
|  | 843 | 4 | 1 | 1K |  |
| CB | 33240 | 3 | 3 | 60K | 12 weeks |
|  | 5540 | 3 | 3 | 10K |  |
|  | 554 | 3 | 1 | 1K |  |
| 32y M BM | 34260 | 3 | 3 | 60K |  |
|  | 5710 | 3 | 2 | 10K |  |
|  | 571 | 3 | 0 | 1K |  |
| 52y F BM | 50580 | 4 | 4 | 60K |  |
|  | 8430 | 4 | 4 | 10K |  |
|  | 843 | 4 | 1 | 1K |  |
| 78y F BM | 43440 | 3 | 3 | 60K |  |
|  | 7240 | 3 | 3 | 10K |  |
|  | 724 | 3 | 1 | 1K |  |
| CB | 33240 | 2 | 2 | 60K | 20 weeks |
|  | 5540 | 3 | 3 | 10K |  |
|  | 554 | 3 | 2 | 1K |  |
| 52y F BM | 50580 | 3 | 3 | 60K |  |
|  | 8430 | 2 | 0 | 10K |  |
|  | 843 | 3 | 0 | 1K |  |

| **EXPT2** | | | | | |
| --- | --- | --- | --- | --- | --- |
| **sample type** | **#CD34+ cells transplanted** | **# mice tested** | **#responders** | **Cell dose** | **Time point** |
| CB | 33000 | 2 | 2 | 60K | 4 weeks |
|  | 5500 | 3 | 2 | 10K |  |
| 21y F BM | 48660 | 3 | 3 | 60K |  |
| 57y M BM | 45540 | 3 | 3 | 60K |  |
|  | 7590 | 3 | 3 | 10K |  |
|  | 759 | 3 | 0 | 1K |  |
| 79y F BM | 51840 | 3 | 3 | 60K |  |
|  | 8640 | 3 | 3 | 10K |  |
|  | 864 | 3 | 2 | 1K |  |
| CB | 33000 | 3 | 3 | 60K |  |
|  | 5500 | 3 | 3 | 10K |  |
|  | 550 | 3 | 0 | 1K |  |
| 21y F BM | 48660 | 3 | 3 | 60K | 12 weeks |
|  | 8110 | 3 | 2 | 10K |  |
|  | 811 | 3 | 0 | 1K |  |
| 57y M BM | 45540 | 3 | 3 | 60K |  |
|  | 7590 | 3 | 3 | 10K |  |
|  | 759 | 3 | 3 | 1K |  |
| 79y F BM | 51840 | 3 | 3 | 60K |  |
|  | 8640 | 3 | 3 | 10K |  |
|  | 864 | 3 | 2 | 1K |  |
| CB | 33000 | 3 | 3 | 60K | 20 weeks |
| 21y F BM | 48660 | 3 | 1 | 60K |  |
| 79y F BM | 51840 | 3 | 3 | 60K |  |
|  | 8640 | 3 | 2 | 10K |  |
|  | 864 | 3 | 0 | 1K |  |

| **EXPT3** | | | | | |
| --- | --- | --- | --- | --- | --- |
| **sample type** | **#CD34+ cells transplanted** | **# mice tested** | **#responders** | **Cell dose** | **Time point** |
| CB | 53640 | 3 | 3 | 60K | 4 weeks |
|  | 894 | 2 | 1 | 1K |  |
| 30y M BM | 21120 | 3 | 3 | 60K |  |
|  | 3520 | 3 | 1 | 10K |  |
|  | 352 | 3 | 0 | 1K |  |
| 55y F BM | 54300 | 3 | 3 | 60K |  |
|  | 9050 | 3 | 2 | 10K |  |
|  | 905 | 3 | 0 | 1K |  |
| 83y M BM | 75200 | 3 | 3 | 100K |  |
|  | 45120 | 3 | 3 | 60K |  |
|  | 7520 | 3 | 2 | 10K |  |
|  | 752 | 3 | 0 | 1K |  |
| CB | 53640 | 3 | 3 | 60K | 12 weeks |
| 30y M BM | 21120 | 4 | 3 | 60K |  |
|  | 3520 | 3 | 2 | 10K |  |
|  | 352 | 3 | 0 | 1K |  |
| 55y F BM | 54300 | 3 | 3 | 60K |  |
|  | 9050 | 3 | 3 | 10K |  |
|  | 905 | 3 | 1 | 1K |  |
| 83y M BM | 45120 | 3 | 3 | 60K |  |
|  | 7520 | 3 | 3 | 10K |  |
|  | 752 | 3 | 1 | 1K |  |
| CB | 53640 | 3 | 3 | CB | 20 weeks |
| 55y F BM | 54300 | 2 | 2 | 60K |  |
|  | 9050 | 3 | 2 | 10K |  |
| 83y M BM | 45120 | 3 | 3 | 60K |  |
|  | 7520 | 3 | 3 | 10K |  |
|  | 752 | 3 | 1 | 1K |  |

| **EXPT4** | | | | | |
| --- | --- | --- | --- | --- | --- |
| **sample type** | **#CD34+ cells transplanted** | **# mice tested** | **#responders** | **Cell dose** | **Time point** |
| CB | 50580 | 3 | 3 | 60K | 4 weeks |
| 32y F BM | 37800 | 3 | 3 | 60K |  |
|  | 6300 | 2 | 2 | 10K |  |
|  | 630 | 3 | 0 | 1K |  |
| 30y F BM | 31980 | 3 | 2 | 60K |  |
|  | 5330 | 2 | 0 | 10K |  |
|  | 533 | 2 | 0 | 1K |  |
| 78y F BM | 48060 | 3 | 3 | 60K |  |
|  | 8010 | 2 | 0 | 10K |  |
|  | 801 | 3 | 0 | 1K |  |
| 77y F BM | 41580 | 3 | 3 | 60K |  |
|  | 6930 | 3 | 3 | 10K |  |
|  | 693 | 3 | 0 | 1K |  |
| CB | 50580 | 3 | 3 | CB | 12 weeks |
| 77y F BM | 41580 | 3 | 3 | 60K |  |
|  | 693 | 3 | 3 | 1K |  |
| CB | 50580 | 3 | 3 | 60K | 20 weeks |
| 32y F BM | 37800 | 2 | 2 | 60K |  |
|  | 6300 | 3 | 1 | 10K |  |
|  | 630 | 3 | 0 | 1K |  |
| 30y F BM | 31980 | 3 | 1 | 60K |  |
|  | 5330 | 3 | 2 | 10K |  |
|  | 533 | 1 | 0 | 1K |  |
| 78y F BM | 48060 | 3 | 3 | 60K |  |
|  | 8010 | 2 | 0 | 10K |  |
|  | 801 | 3 | 1 | 1K |  |
| 77y F BM | 41580 | 3 | 2 | 60K |  |
|  | 6930 | 3 | 1 | 10K |  |
|  | 693 | 3 | 0 | 1K |  |

| **Confidence intervals for 1/(stem cell frequency)** | | | | | | | | | |
| --- | --- | --- | --- | --- | --- | --- | --- | --- | --- |
| **Group** | **4 weeks** | | | **12 weeks** | | | **20 weeks** | | |
|  | **Lower** | **Estimate** | **Upper** | **Lower** | **Estimate** | **Upper** | **Lower** | **Estimate** | **Upper** |
| CB | 4328 | 1647 | 626 | 4409 | 1694 | 651 | 2158 | 504 | 118 |
| Young BM | 23582 | 11944 | 6050 | 19743 | 10052 | 5118 | 82481 | 36280 | 15958 |
| Mid-age BM | 9207 | 4487 | 2187 | 2832 | 1208 | 515 | 37792 | 14537 | 5592 |
| Old BM | 10792 | 5574 | 2879 | 2419 | 1097 | 497 | 22988 | 12247 | 6525 |

| **Pairwise tests for differences in stem cell frequencies** | | | | | | | | |
| --- | --- | --- | --- | --- | --- | --- | --- | --- |
| **4 weeks** | | | **12 weeks** | | | **20 weeks** | | |
| **Group 1** | **Group 2** | **P value** | **Group 1** | **Group 2** | **P value** | **Group 1** | **Group 2** | **P value** |
| Mid-age BM | Old BM | 0.666 | Young BM | Mid-age BM | 8.95E-05 | Young BM | Mid-age BM | 0.151 |
| Mid-age BM | Young BM | 0.0579 | Young BM | Old BM | 2.73E-05 | Young BM | Old BM | 0.0197 |
| Mid-age BM | CB | 0.0722 | Young BM | CB | 0.00437 | Young BM | CB | 7.03E-07 |
| Old BM | Young BM | 0.112 | Mid-age BM | Old BM | 0.87 | Mid-age BM | Old BM | 0.771 |
| Old BM | CB | 0.0226 | Mid-age BM | CB | 0.621 | Mid-age BM | CB | 0.000438 |
| Young BM | CB | 0.000355 | Old BM | CB | 0.514 | Old BM | CB | 0.000224 |
