# Supplementary material for: Molecular and phenotypic blueprint of human hematopoiesis links proliferation stress to stem cell aging
Source: J Exp Med. 2025 Dec 30;223(2):e20251805. doi: 10.1084/jem.20251805 (PMC13248933; doi:10.1084/jem.20251805)
Supplement: Table S3 — shows annotated clusters per dataset. [file jem_20251805_tables3.docx]

**Table S3. Annotated clusters per dataset**

|  | **Ainciburu et al** | | **Li et al** | **Mende et al** | | **Quaranta et al** | | **Setty et al** | |
| --- | --- | --- | --- | --- | --- | --- | --- | --- | --- |
| HSC-MPP | 17476 | 2830 | | 1878 | 7666 | | 1973 | |  |
| Cycling-HSC | 33 | 24 | | 0 | 814 | | 9 | |  |
| CMP | 3661 | 276 | | 153 | 1367 | | 505 | |  |
| Myelo/Lympho-CMP | 10632 | 1101 | | 1071 | 4513 | | 2360 | |  |
| CMP-GMP | 5524 | 721 | | 422 | 2355 | | 1363 | |  |
| GP | 5029 | 670 | | 267 | 1091 | | 1055 | |  |
| MDP | 8729 | 1249 | | 373 | 2036 | | 2896 | |  |
| MLP | 929 | 76 | | 96 | 215 | | 164 | |  |
| PreB | 2343 | 756 | | 175 | 627 | | 2484 | |  |
| PreNK | 1379 | 453 | | 28 | 288 | | 369 | |  |
| MEP | 4783 | 391 | | 232 | 1443 | | 742 | |  |
| ImmEry | 4266 | 454 | | 267 | 1697 | | 985 | |  |
| MatEry | 6266 | 806 | | 330 | 1673 | | 1357 | |  |
| MKP | 1655 | 236 | | 116 | 453 | | 256 | |  |
| BEM cells | 2206 | 219 | | 84 | 504 | | 259 | |  |

**Annotated clusters per donor**

|  | **Ainciburu_elderly1** | **Ainciburu_elderly2** | **Ainciburu_elderly3** |
| --- | --- | --- | --- |
| HSC-MPP | 1769 | 8327 | 3734 |
| Cycling-HSC | 3 | 9 | 9 |
| CMP | 337 | 628 | 946 |
| Myelo/Lympho-CMP | 992 | 2393 | 2060 |
| CMP-GMP | 394 | 881 | 1007 |
| GP | 343 | 658 | 1000 |
| MDP | 588 | 637 | 1431 |
| MLP | 40 | 35 | 249 |
| PreB | 113 | 237 | 411 |
| PreNK | 26 | 59 | 97 |
| MEP | 885 | 1237 | 1022 |
| ImmEry | 465 | 815 | 1051 |
| MatEry | 860 | 1158 | 1433 |
| MKP | 193 | 358 | 581 |
| BEM cells | 233 | 443 | 426 |

|  | **Ainciburu_young1** | **Ainciburu_young2** | **Ainciburu_young3** | **Ainciburu_young4** | **Ainciburu_young5** |
| --- | --- | --- | --- | --- | --- |
| HSC-MPP | 244 | 543 | 616 | 1210 | 1033 |
| Cycling-HSC | 0 | 4 | 0 | 2 | 6 |
| CMP | 68 | 120 | 274 | 442 | 846 |
| Myelo/Lympho-CMP | 364 | 674 | 835 | 1458 | 1856 |
| CMP-GMP | 139 | 371 | 560 | 977 | 1195 |
| GP | 177 | 370 | 471 | 1001 | 1009 |
| MDP | 529 | 1112 | 1339 | 1401 | 1692 |
| MLP | 26 | 33 | 48 | 37 | 461 |
| PreB | 153 | 203 | 317 | 183 | 726 |
| PreNK | 124 | 165 | 279 | 58 | 571 |
| MEP | 131 | 189 | 201 | 509 | 609 |
| ImmEry | 96 | 222 | 273 | 518 | 826 |
| MatEry | 200 | 392 | 353 | 732 | 1138 |
| MKP | 34 | 63 | 63 | 172 | 191 |
| BEM cells | 78 | 94 | 181 | 265 | 486 |

|  | **Li_BM1** | **Li_BM3** | **Li_GR90** | **Li_GR91** | **Li_GR92** | **Li_GR93** | **Li_GR94** |
| --- | --- | --- | --- | --- | --- | --- | --- |
| HSC-MPP | 594 | 537 | 224 | 182 | 457 | 323 | 513 |
| Cycling-HSC | 2 | 3 | 8 | 5 | 1 | 1 | 4 |
| CMP | 92 | 64 | 16 | 13 | 34 | 31 | 26 |
| Myelo/Lympho-CMP | 411 | 242 | 74 | 28 | 131 | 71 | 144 |
| CMP-GMP | 259 | 198 | 44 | 23 | 67 | 47 | 83 |
| GP | 283 | 215 | 40 | 8 | 49 | 27 | 48 |
| MDP | 677 | 364 | 43 | 9 | 58 | 42 | 56 |
| MLP | 28 | 26 | 6 | 1 | 6 | 3 | 6 |
| PreB | 256 | 421 | 29 | 7 | 17 | 9 | 17 |
| PreNK | 168 | 251 | 21 | 0 | 4 | 3 | 6 |
| MEP | 91 | 64 | 28 | 38 | 59 | 45 | 66 |
| ImmEry | 108 | 67 | 15 | 45 | 49 | 80 | 90 |
| MatEry | 166 | 128 | 25 | 118 | 77 | 130 | 162 |
| MKP | 34 | 30 | 19 | 53 | 19 | 44 | 37 |
| BEM cells | 91 | 63 | 10 | 6 | 13 | 17 | 19 |

|  | **Mende_DOD1** |
| --- | --- |
| HSC-MPP | 1878 |
| Cycling-HSC | 0 |
| CMP | 153 |
| Myelo/Lympho-CMP | 1071 |
| CMP-GMP | 422 |
| GP | 267 |
| MDP | 373 |
| MLP | 96 |
| PreB | 175 |
| PreNK | 28 |
| MEP | 232 |
| ImmEry | 267 |
| MatEry | 330 |
| MKP | 116 |
| BEM cells | 84 |

|  | **Quaranta_B** | **Quaranta_BMC** | **Quaranta_BMfem** | **Quaranta_HD14** |
| --- | --- | --- | --- | --- |
| HSC-MPP | 83 | 1378 | 1455 | 4750 |
| Cycling-HSC | 7 | 0 | 6 | 801 |
| CMP | 32 | 390 | 457 | 488 |
| Myelo/Lympho-CMP | 115 | 1070 | 1452 | 1876 |
| CMP-GMP | 68 | 579 | 637 | 1071 |
| GP | 58 | 333 | 398 | 302 |
| MDP | 124 | 680 | 716 | 516 |
| MLP | 11 | 39 | 100 | 65 |
| PreB | 20 | 123 | 307 | 177 |
| PreNK | 18 | 40 | 174 | 56 |
| MEP | 34 | 369 | 337 | 703 |
| ImmEry | 20 | 531 | 357 | 789 |
| MatEry | 21 | 738 | 522 | 392 |
| MKP | 5 | 227 | 90 | 131 |
| BEM cells | 14 | 124 | 211 | 155 |

|  | **Setty_BM2** | **Setty_BM3** |
| --- | --- | --- |
| HSC-MPP | 842 | 1131 |
| Cycling-HSC | 5 | 4 |
| CMP | 203 | 302 |
| Myelo/Lympho-CMP | 853 | 1507 |
| CMP-GMP | 391 | 972 |
| GP | 335 | 720 |
| MDP | 955 | 1941 |
| MLP | 87 | 77 |
| PreB | 396 | 2088 |
| PreNK | 52 | 317 |
| MEP | 297 | 445 |
| ImmEry | 427 | 558 |
| MatEry | 834 | 523 |
| MKP | 82 | 174 |
| BEM cells | 95 | 164 |
