# Supplementary material for: Molecular and phenotypic blueprint of human hematopoiesis links proliferation stress to stem cell aging
Source: J Exp Med. 2025 Dec 30;223(2):e20251805. doi: 10.1084/jem.20251805 (PMC13248933; doi:10.1084/jem.20251805)
Supplement: Table S4 — shows sample information for bulk ATAC-seq NMF analysis. [file jem_20251805_tables4.docx]

**Table S4. Sample information for bulk ATAC-seq NMF analysis**

| **Population** | **BM source** | **Age** | **Gender** | **Group** |
| --- | --- | --- | --- | --- |
| CD49f+ HSC | Lonza | 21 | F | Young |
| CD49f- MPP |  |  |  |  |
| GMP |  |  |  |  |
| CMP |  |  |  |  |
| MEP |  |  |  |  |
| MLP |  |  |  |  |
| CD49f+ HSC | Lonza | 28 | F |  |
| CD49f- MPP |  |  |  |  |
| GMP |  |  |  |  |
| CMP |  |  |  |  |
| MEP |  |  |  |  |
| MLP |  |  |  |  |
| CD49f+ HSC | Mexico | 27 | M |  |
| CD49f- MPP |  |  |  |  |
| GMP |  |  |  |  |
| CMP |  |  |  |  |
| CD49f- MPP | Mexico | 52 | F | Old |
| GMP |  |  |  |  |
| CMP |  |  |  |  |
| MLP |  |  |  |  |
| MEP |  |  |  |  |
| CD49f+ HSC | Mexico | 58 | F |  |
| GMP |  |  |  |  |
| CD49f+ HSC | Mexico | 61 | F |  |
| CD49f- MPP |  |  |  |  |
| GMP |  |  |  |  |
| CMP |  |  |  |  |
| CD49f+ HSC | Mexico | 79 | F |  |
| CD49f- MPP |  |  |  |  |
| GMP |  |  |  |  |
| MEP |  |  |  |  |
