# Supplementary material for: Molecular and phenotypic blueprint of human hematopoiesis links proliferation stress to stem cell aging
Source: J Exp Med. 2025 Dec 30;223(2):e20251805. doi: 10.1084/jem.20251805 (PMC13248933; doi:10.1084/jem.20251805)
Supplement: Table S7 — shows RT-qPCR primers. [file jem_20251805_tables7.docx]

**Table S7. RT-qPCR primers**

| **Primers target** | **Optimal concentration of primers** | **Sequence FW** | **Sequence REV** |
| --- | --- | --- | --- |
| GUSB | 350 nM | CTGACACCTCCAAGTATCCCAAG | GTCGTGTACAGAAGTACAGACCGC |
| IL1A | 120 nM | GGTTGAGTTTAAGCCAATCCA | TGCTGACCTAGGCTTGATGA |
| IL1B | 100 nM | GCTCAAGTGTCTGAAGCAGCC | CAGCTTCAAAGAACAAGTCATCCT |
| p21 | 90 nM | CAGCATGACAGATTTCTACCACTC | CTCGCGCTTCCAGGACTG |
| p16 | 160 nM | CCAACGCACCGAATAGTTACG | GCGCTGCCCATCATCATG |
| IL8 | 100 nM | CATCTCACTGTGTGTAAACATGAC | CCTTGGCAAAACTGCACCTTCAC |
| IL6 | 130 nM | GATTCAATGAGGAGACTTGCCTGG | CTCACTACTCTCAAATCTGTTCTGG |
| TNFalfa | 100 nM | CCAGGGACCTCTCTCTAATCAGC | GGTTTGCTACAACATGGGCTAC |
| MCP1 | 150 nM | CTGTGATCTTCAAGACCATTGTG | AGTTTGGGTTTGCTTGTCCAG |
